# Fishers’ knowledge on the coast of Brazil

**DOI:** 10.1186/s13002-016-0091-1

**Published:** 2016-06-01

**Authors:** Alpina Begossi, Svetlana Salivonchyk, Priscila F. M. Lopes, Renato A. M. Silvano

**Affiliations:** Capesca, NEPA, UNICAMP, Av. Albert Einstein 291, CEP 13083-852 Campinas, SP Brazil; Fisheries and Food Institute, http://www.fisheriesandfood.org; Ecomar, Unisanta, Rua Cesário Mota, 08, CEP 11045-040 Santos, SP Brazil; Institute for Nature Management, National Academy of Sciences of Belarus, 10 Fr. Skaryna Street, Minsk, 220114 Minsk, Belarus; Fishing Ecology, Management and Economics Group (FEME), Department of Ecology, Federal University of Rio Grande do Norte (UFRN), Natal, RN 59078-900 Brazil; Universidade Federal do Rio Grande do Sul (UFRGS), Department of Ecology, CP 15007, 91501-970 Porto Alegre, RS Brazil

## Abstract

**Background:**

Although fishers’ knowledge has been recently considered into management programmes, there is still the need to establish a better understanding of fishers’ perceptions and cognition. Fishers can provide novel information on the biology and ecology of species, which can potentially be used in the management of fisheries. The knowledge fishers have and how they classify nature is empirically based. It is common, for example, to observe that fishers’ taxonomy is often represented by the generic level, one of the hierarchical categories of folk classification that is somewhat analogous to the *Linnean* genus, as it groups organisms of a higher rank than the folk species.In this study we compiled the knowledge fishers have on local fish, such as their folk names, diet and habitat.

**Methods:**

Five coastal communities widely distributed along the Brazilian coast were studied: two from the northeast (Porto Sauípe and Itacimirim, in Bahia State, n of interviewees = 34), two from the southeast (Itaipu at Niterói and Copacabana at Rio de Janeiro, Rio de Janeiro State, *n* = 35) and one from the south coast (Pântano do Sul, in Santa Catarina State, *n* = 23). Fish pictures were randomly ordered and the same order was presented to all interviewees (*n* = 92), when they were then asked about the species name and classification and its habitat and diet preferences.

**Results:**

Fishers make clusters of fish species, usually hierarchically; fishers of the coast of Brazil use mostly primary lexemes (generic names) to name fish; and fishers did not differentiate between scientific species, since the same folk generic name included two different scientific species. Fishers provide information on species to which there is scarce or no information on diet and habitat, such as *Rhinobatos percellens* (chola guitarfish, arraia viola or cação viola)*, Sphoeroides dorsalis (*marbled puffer*,* baiacu), *Mycteroperca acutirostris* (comb grouper, badejo) and *Dasyatis guttata* (longnose stingray, arraia, arraia manteiga).

**Conclusions:**

fishers’ knowledge on fish diet and fish habitat can be strategic to management, since their knowledge concentrates on the fishery target species, which are the ones under higher fishing pressure. Besides, fishers showed to have knowledge on species still poorly known to science.

**Electronic supplementary material:**

The online version of this article (doi:10.1186/s13002-016-0091-1) contains supplementary material, which is available to authorized users.

## Background

Ethnobiology includes the labelling of organisms (ethnotaxonomy), their recognition by diagnostic characteristics (*diagnostic characters*) and their cultural classification, which implies their grouping according to a set of criteria following a classification system [1]. The understanding of clusters of organisms, the criteria of group formation, along with higher hierarchical forms, such as ‘life forms’, forms a body of theory that helps understand the human classification of nature and human cognition concerning nature ‘discontinuities’ [2]. A study on Brazilian fishers [3] has shown that fishers form fish groups, hierarchically, based on morphology, ecology and fish behaviour. Among the multiple questions raised in ethnobiological studies, one approaches how the knowledge people have about nature varies with culture, experience, or expertise [4]. Another question addresses if human perception is selective, by focusing better on more salient items (or organisms). Salient organisms can be beautiful, colourful, big or cultural relevant organisms, among other attributes [5]. Therefore, the way people name and classify nature is also associated to human perception, expression and cognition. It has been shown, for example, that people tend to classify nature often up to a genus level (primary lexemes), represented by generic names [1].

The reason why multiple ethnobiology studies have been done with fishers is exactly due to their direct contact with a wide range of organisms, and especially in tropical countries, where there is a high diversity of species. Specifically, the ethnotaxonomy and classification systems of fish by artisanal fishers have been widely studied, including several studies in coastal Brazil [3, 6–14]. Understanding how fishers name fish, along with other information they have about the ecology and biology of species, could help us grasp part of the human cognition. Human perception abilities, such as when observing nature, is an important attribute and might be concentrated on more salient organisms (e.g.: beautiful, colourful useful, big organisms). Such understanding could also help us in other ways. For example, target species seem to be those that fishers focus their daily attention [3, 15]. If we conclude that fishers know considerably more about target species than about non-target ones, consequences for managing fisheries are manifold. For example, fishers’ knowledge regarding fish diversity could be useful to monitor temporal or spatial changes in fish species composition or abundance [16], as well as to compare fishers’ knowledge with official fish landings statistics [17]. Furthermore, fishers’ knowledge on ethnotaxonomy could improve inventories of fish diversity, at least for target species [3].

Ethnoecology, or more specifically ethnoichthyology, meaning the knowledge fishers have about fish biology and ecology, has also been studied in Brazil. Ethnoichthyology is a subdivision of ethnozoology [18]. Besides more basic research questions from ethnozoology and ethnoichthyology, the simple need of information about fish is of great importance: in particular, the lack of information on fish from the Brazilian coast is striking, also because the collection of data on fish landings on this coast is unreliable. Some studies have focused on fishers’ knowledge of spawning and migration of coastal fishes [19]. Some other studies have also examined fishers’ knowledge of trophic interactions in SE Brazil [20–22]. Particularly, there is increasing interest in having fishers contribute their knowledge to the management of fisheries [23, 24].

However, studies are generally local or regional and cover specific fishing communities. Collecting data is time-consuming, and there are few studies that encompass larger areas to permit broader comparative analysis of fishers’ knowledge [18, 25, 26]. We thus developed an ethnobiological study encompassing several fishing communities in major coastal areas of Brazil (northeast, southeast and south). We also developed a comparative method: instead of using locally landed data, we used identical pictures of fish at all studied sites.

Our objectives were to obtain knowledge on general patterns of fishers’ folk nomenclature and classification processes. We also tested with quantitative data two hypotheses regarding factors influencing fishers’ knowledge on coastal fish species: 1) fishers’ knowledge will be positively related to fish size (see also [5]) so larger fish would be better known; and 2) fishers’ knowledge will be positively related to economic importance of fish species, so fish with high economic importance would be better known. Finally, we suggest these data are worth to environmental agencies and researchers, in order to improve the management of target species.

## Methods

**Fig. 1:**
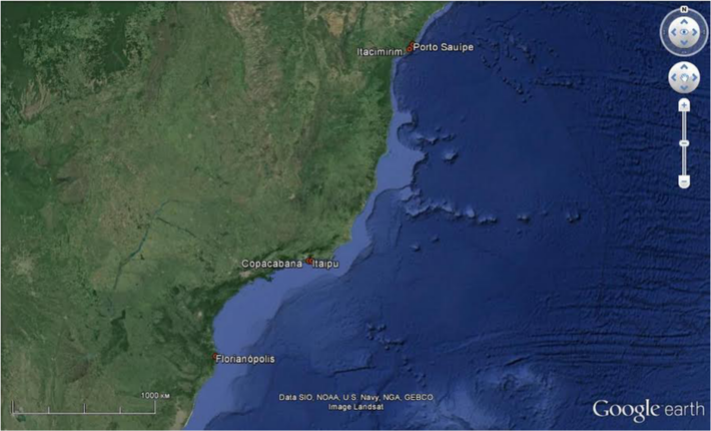
Map of the communities studied in the coast of Brazil. Northeast: Itacimirim and Porto do Sauípe, Bahia State; Southeast: Copacabana and Itaipu, Rio de Janeiro State; South: Florianópolis, Santa Catarina State

Five coastal communities were studied between 2004 and 2005: two from the northeast (Porto Sauípe and Itacimirim, in Bahia State), two from the southeast (Itaipu at Niterói and Copacabana at Rio de Janeiro, Rio de Janeiro State) and one from the south coast (Pântano do Sul, Florianópolis, in Santa Catarina State) (Fig. 1). These sites were visited in earlier studies [27] (Fig. 1).

**Fig. 2:**
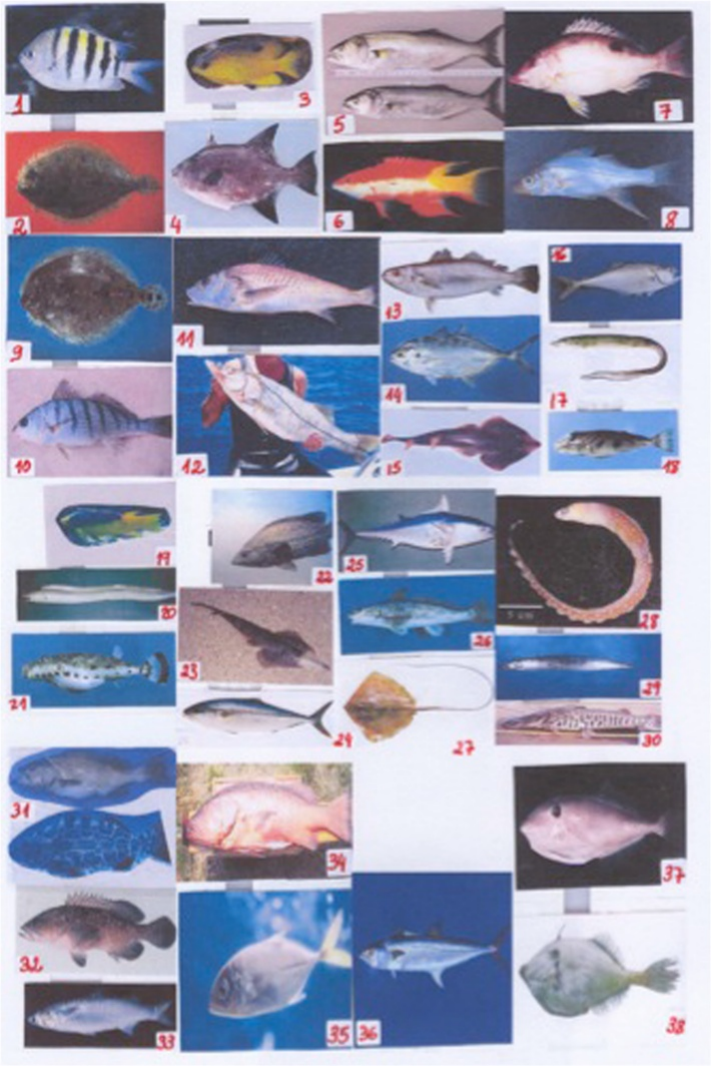
Methods: pictures of the fish showed to fishers with the order number

We chose the fish species to be studied according to their commonness, rarity, and usefulness (for example, eaten and sold, among others.). This information was obtained from fish landings we had from earlier research at these areas [27]. We expected to find more information on common species (target species) rather than on rare ones. For that reason we used prior information on landings to identify the target and rare species. Probably we could expect more knowledge on salient species; but target species, being also salient, are testable. We expanded an earlier study in which 24 pictures were shown to fishers at some sites in Brazil [3, 12]. In the present one, we showed pictures of 38 fish species to fishers. The fish pictures were randomly ordered, and the same order was presented to all interviewees (Fig. 2). One of the pictures was a control (a freshwater fish of no occurrence at the study sites, the *Pseudoplatystoma fasciatum*, Pimelodidae).

We only interviewed fishers who were over 35 years old and who had lived in the community being studied for the last 20 years. We conducted interviews at landing points and at fishers’ residences. Information regarding fishing activities and on fishers, is available [27]. The interview protocol, after the verbal consent of the interviewer, included the fish identification of the picture (folk name), knowledge of the fish diet, of the fish habitat, among other questions. The last protocol included placing the fish (based on the order number) on a table, or at the floor, and asking the fisher to form groups as they perceived them (for example, groups of fish considered by fishers to be ‘cousins’ or ‘relatives’). The questionnaires are deposited in the FIFO’s archives, labeled PEMVIMSA 005 (Fisheries and Food Institute, located at Ecomar, Unisanta, Santos, SP, Brazil). Interviews included 34 fishers from Bahia State (22 from Porto Sauípe and 12 from Itacimirim), 35 from Rio de Janeiro State (12 from Itaipu, Niterói, and 23 from Copacabana, Rio) and 23 from Santa Catarina State (Pântano do Sul, Florianopolis), with a total of 92 fishers.

We compared our data on fishers’ knowledge about fish biology and classification with the scientific literature that is available and synthesized in Fishbase [28], amongst others, which are references cited along the text (see also Additional file 1).

We tested the two hypotheses regarding the influences of fish size (hypothesis 1) and value (hypothesis 2) on the local fishers’ knowledge. These hypotheses were tested by using two measures of fishers’ knowledge (the dependent variables in the analyses): number of doubts (when the fisherman answered ‘I do not know’) and homogeneity of identification of fish species. Number of doubts was adopted as an inverse indicator of knowledge: the higher the number of doubts, the lesser the knowledge, following an approach adopted in previous ethnobiological studies [3, 18, 29]. The homogeneity of answers was measured as the total number of interviewed fishers who cited the most mentioned name for the fish species in each studied community, calculated from data in Table 1. This is a direct indicator of knowledge: a higher number of fishers mentioning the same name for a fish indicates that this fish is better known, as it shows higher homogeneity within the population and is more readily recognized by fishers [3]. The economic value of fish species (independent variable) was assigned based on previous studies on fish preferences [26, 30] and on the expertise of the authors. Each species was classified as low, medium or high priced fish. The other independent variable, fish size, was gathered from the Fishbase website [28], measured as maximum reported length for each species. We checked normality of the continuous variables (fish size, number of doubts, number of fishers reporting main fish name) and the variable number of doubts was log transformed to perform the statistical analyses. The number of doubts (Log transformed) and number of fishers mentioning the main fish name were compared among three economic categories (high, medium low) of fish species through an Analysis of Variance (ANOVA). The number of doubts (Log transformed) and number of fishers mentioning the main fish name were related to fish size (cm) through linear regression analyses. We checked the relation of these two dependent variables [number of doubts (Log transformed) and number of fishers mentioning the main fish name] through Pearson correlation analysis.

**Table 1:** Folk names given by fishermen of the coast of Brazil (*n* = 92)

Species	Porto Sauípe BAHIA (*n* = 22)	Itacimirim BAHIA (*n* = 12)	Northeast: Itacimirim & Porto Sauipe (*n* = 34)	Itaipu NITEROI (*n* = 12)	Copacabana RIO DE JANEIRO RJ (*n* = 23)	Southeast: Copacabana& Itaipu (*n* = 35)	Southern: Florianópolis (*n* = 23)
*1. Abudefduf* *saxatilis*	Capiacaba (18) Corro (2)	Capiacaba (5) Dnk (3)	Capiacaba (23) Dnk (4)	Sargento = 11(8 + 3) (Sargento mouro = 3)	Sargento (12) Sargo = 4(3 + 1) (Sargo de dente = 1) Dnk (3)	Sargento = 23 (20 + 3) (Sargento mouro = 3) Sargo = 4(3 + 1) (Sargo de dente = 1) Dnk (3)	Sargo or Salgo = 17 (2 + 15) (Salgo (Sargo) de dente =7 Salgo (Salguinho) de dentro = 7 Sargo de beiço = 1)
*2. Bothus* *ocellatus*	Aramaca’ (21)	Aramaca’ (6) Soio (6) Linguado (4)	Aramaca’ (27) Soio (7) Linguado (5) Tapa (3)	Linguado (12)	Linguado (20) Solha = 7 (6 + 1) (Solha pequeno = 1)	Linguado (32) Solha = 7 (6 + 1) (Solha pequeno = 1)	Linguado = 22 (19 + 3) (Linguado chinelo = 1 Linguado da lama = 1 Linguado de areia = 1)
*3. Stegastes* *leucostictus*	Pantucano or Plantucano (8) Dnk (7)	Soldado (2) Dnk (4)	Pantucano or Plantucano (8) Ze pereira (4) Dnk (11)	Marimba ouro (1)	Budião (2) Dnk (15)	Dnk (15)	Dnk (17)
*4. Canthidermis* *sufflamen*	Piraca’ (8) Capado branco (7) Capado reis (3) Dnk (3)	Capado branco (4) Piraca’ (3) Dnk (2)	Capado branco (11) Piraca’ (11) Porco (4) Capado reis (3) Peixe rato (3) Dnk (5)	Porco (7) Cangulo (6)	Cangulo (15) Porco (4)	Cangulo (21) Porco (11) Perua (3)	Peixe porco = 20 (18 + 2) (Peixe porco legítimo = 1 Peixe porco branco = 1) Cangulu or Cangula (7) Porco (3)
*5. Pomatomus* *saltatrix*	Enchova (13) Dnk (4)	Enchova (7) Dnk (3)	Enchova (20) Pescada (3) Dnk (7)	Enchova (12)	Enchova (22)	Enchova (34)	Anchova or Anchoveta = 19 (17 + 2) (Anchova marisqueira = 1 Anchova da pedra = 1) Enchova or Enchoveta = 4 (3 + 1) (Enchova do corso = 1)
*6. Bodianus* *pulchellus*	Plantucano or Pantucano (15) Ze pereira (3)	Soldado (5) Dnk (3)	Plantucano or Pantucano (15) Soldado (5) Ze pereira (3) Dnk (4)	Jaguriça or Jaguruça (2)	Budião (3) Dnk (11)	Budião (4) Dnk (11)	Dnk (16)
*7. Lutjanus* *synagris*	Arioco’ (16)	Arioco’ (4)	Arioco’ (20) Carapitanga (4) Quatinga (4) Vermelho (3)	Vermelho =7 (1 + 6) (Vermelho caranho = 4 Vermelho cioba = 2)	Vermelho = 11 (1 + 10) (Vermelho caranha =4 Vermelho cioba = 4 Vermelho-henrique = 2) Dnk (7)	Vermelho = 18 (2 + 16) (Vermelho cioba = 6 Vermelho caranha =4 Vermelho caranho =4 Vermelho-henrique = 2) Dnk (7)	Cocoroca = 15 (9 + 6) (Cocoroca da boca larga = 4 Cocoroca do norte = 1 Cocoroca do costão = 1) Vermelho (4)
*8. Centropomus* *parallelus*	Robalo = 22 (11 + 11) (Robalo corcunda = 6, Robalo furao = 4 Robalo coruja = 1)	Robalo = 11 (7 + 4) (Robalo barriga-mole = 1 Robalo esplamado = 1 Robalo verdadeiro = 1 Robalo’loco = 1)	Robalo =33(18 + 15) (Robalo corcunda = 6 Robalo furao = 4 Robalo barriga-mole = 1 Robalo coruja = 1 Robalo esplamado = 1 Robalo verdadeiro = 1 Robalo’loco = 1)	Robalo (12)	Robalo =23(20 + 3) (Robalo peba = 2 Robalo flecha = 1) Camorim (3)	Robalo =35(32 + 3) (Robalo peba = 2 Robalo flecha = 1) Camorim (3)	Robalo (23)
*9. Bothus* *robinsi*	Aramaca’ (16) Soio (3) Tapa or Taipa (3)	Soio (6) Tapa (4) Aramaca’ (3) Linguado (3)	Aramaca’ (19) Soio (9) Tapa (6) Linguado (4)	Linguado (12)	Linguado = 18 (17 + 1) (Linguado cascalho = 1) Solha or Soia (5)	Linguado (29) Solha or Soia (6)	Linguado = 23 (20 + 3) (Linguado branco = 1 Linguado chinelo = 1 Linguado de areia = 1)
*10. Umbrina* *coroides*	Roncador =22 (3 + 19) (Roncador judeus =17 Roncad or branco = 1 Roncador cachorro = 1)	Roncador branco (5) Roncador (2)	Roncador =29 (5 + 24) (Roncador judeus =17 Roncador branco = 6 Roncador cachorro = 1)	Riscadinha (8)	Roncador (9) Cangua or Tangua (7) Dnk (4)	Roncador (9) Riscadinha(o) (9) Cangua or Tangua (7) Corvina (3) Dnk (4)	Maria luiza (8) Roncador = 6 (4 + 2) (Roncador do norte = 2) Papa terra = 5 (3 + 2) (Papa terra do assobio = 1 Papa terra de assorio = 1) Curvina = 3(1 + 2) (Curvina riscada = 1 Curvina pintada = 1)
*11. Micropogonias* *furnieri*	Papa-terra (13) Corvina (8)	Corvina (4) Dnk (3)	Papa-terra (14) Corvina (12) Dnk (3)	Corvina (12)	Corvina (22)	Corvina (34)	Curvina (17) Curvinota (4) Corvina (3)
*12. Centropomus* *undecimalis*	Robalo = 22 (10 + 12) (Robalo corcunda =5 Robalo cambriacu = 4 Robalo furao = 2 Robalo morcego = 1)	Robalo = 12 (8 + 4) (Robalo corcunda =2 Robalo branco = 1 Robalo cambriacu = 1)	Robalo = 34 (18 + 16) (Robalo corcunda =7 Robalo cambriacu = 5 Robalo furao = 2 Robalo branco = 1 Robalo morcego = 1)	Robalo = 11 (10 + 1) (Robalo flecha =1)	Robalo = 23 (10 + 13) (Robalo flexa =13)	Robalo = 34 (20 + 14) (Robalo flecha =14)	Robalo (20) Dnk (3)
*13. Cynoscion* *jamaicensis*	Samucanga (7) Pescada (3) Dnk (7)	Perna de moça (3) Dnk (7)	Samucanga (7) Pescada (3) Perna de moça (3) Dnk (14)	Guete =5(4 + 1) (Guete de pedra = 1) Pescada (3)	Pescada =9 (3 + 6) (Pescada goete =5 Pescada branca = 1) Perna de moça (4) Dnk (5)	Pescada =12 (6 + 6) (Pescada goete =5 Pescada branca = 1) Guete =5(4 + 1) (Guete de pedra = 1) Perna de moça (4) Dnk (5)	Pescada or Pescadinha = 22 (16 + 6) (Pescada or Pescadinha de bucho = 6)
*14. Caranx* *crysos*	Guaricema (21)	Guaricema (10)	Guaricema (31)	Xerelete (12)	Xerelete (21)	Xerelete (33)	Canarinho (13) Xerelete (12) Manezinho (11) Xaréu (6)
*15. Rhinobatos* *percellens*	Viola (22)	Viola (11)	Viola (33)	Viola (9) Arraia viola (2)	Cação = 12 (3 + 9) (Cação viola = 9) Viola = 8 (6 + 2) (Viola amarela = 2) Arraia viola oor Raia viola (5)	Cação = 13 (3 + 10) (Cação viola = 9 Cação anjo = 1) Viola = 17 (15 + 2) (Viola amarela = 2) Arraia viola oor Raia viola (7)	Viola = 23(22 + 1) (Viola normal = 1)
*16. Oligoplites* *saliens*	Guaibira (17) Solteira (3) Cavaco (3) Dnk (3)	Solteira (9)	Guaibira (17) Solteira (12) Cavaco (4) Dnk (4)	Guaibira (12)	Guaibira (20) Dnk (2)	Guaibira (32)	Guaivira (22)
*17. Conger* *orbignianus*	Caramuru or Caramburu or Camburu = 13 (12 + 1) (Caramuru verde = 1) Mututuca (3)	Caramburu or Caramuru (9)	Caramuru or Caramburu or Camburu = 22 (21 + 1) (Caramuru verde = 1) Mututuca (3) Moreia (3) Dnk (3)	Mussum (4) Congro (3)	Moreia (17)	Moreia (18) Mussum (4) Congro (4)	Cobra = 12 (7 + 5) (Cobra do mar = 5) Moreia (7) Congo = 6 (5 + 1) (Congo da pedra = 1) Dnk (3)
*18. Sphoeroides* *dorsalis*	Baiacu = 20 (14 + 6) (Baiacu guima = 5 Baiacu verde = 1) Guima (3)	Baiacu (3) Baiacu guima (2) Guima (2)	Baiacu = 25 (17 + 8) (Baiacu guima = 7 Baiacu verde = 1) Guima (5)	Baiacu = 11 (8 + 3) (Baiacu arara =3)	Baiacu = 17 (15 + 2) (Baiacu arara = 2) Dnk (3)	Baiacu = 28 (23 + 5) (Baiacu arara = 5) Dnk (3)	Baiacu = 11 (10 + 1) (Baiacu amarelo = 1) Peixe cabra (3) Dnk (4)
*19.Bodianus* *rufus*	Plantucano or Pantucano = 13 (12 + 1) (Plantucano de pedra = 1) Ze pereira or pereira (5)	Soldado (5) Dnk (5)	Plantucano or Pantucano = 13 (12 + 1) (Plantucano de pedra = 1) Soldado (5) Ze pereira or pereira (5) Dnk (7)	Budião = 2 (1 + 1) (Budião papagaio = 1)	Budião = 7(6 + 1) (Budião azul = 1) Dnk (10)	Budião = 9(7 + 2) (Budião azul = 1 Budião papagaio = 1) Dnk (10)	Dnk (14)
*20. Gymnothorax* *funebris*	Caramuru or Caramburu or Camburu (18) Dnk (3)	Caramburu or Caramuru (10) Moreia (4)	Caramuru or Caramburu or Camburu (28) Moreia (6)	Moreia (9)	Moreia = 14 (11 + 3) (Moreia mantega = 1 Moreia preta = 1 Moreia verde = 1) Dnk (3)	Moreia = 23 (20 + 3) (Moreia mantega = 1 Moreia preta = 1 Moreia verde = 1) Dnk (3)	Moreia (9) Cobra = 5 (4 + 1) (Cobra d’água = 1) Dnk (8)
*21. Sphoeroides* *spengleri*	Baiacu = 21 (16 + 5) (Baiacu guima =3 Baiacu do rio = 1 Baiacu pintado = 1)	Baiacu =9(6 + 3) (Baiacu guima = 2 Baiacu de couro = 1)	Baiacu = 30 (22 + 8) (Baiacu guima =5 Baiacu de couro = 1 Baiacu do rio = 1 Baiacu pintado = 1)	Baiacu = 12 (10 + 2) (Baiacu arara =2)	Baiacu = 18 (13 + 5) (Baiacu arara = 3 Baiacu mirim = 1 Baiacu pintado = 1)	Baiacu = 30 (23 + 7) (Baiacu arara = 5 Baiacu mirim = 1 Baiacu pintado = 1)	Baiacu = 18 (12 + 6) (Baiacu amarelo = 6) Biacu (3)
*22. Mycteroperca* *acutirostris*	Badejo (14) Pirambeba (3)	Badejo = 7(6 + 1) (Badejo ferro = 1)	Badejo = 21(20 + 1) (Badejo ferro = 1) Garoupa (4) Pirambeba (4)	Badejo (12)	Badejo = 18 (17 + 1) (Badejo-mira = 1) Cherne (4)	Badejo = 30 (29 + 1) (Badejo-mira = 1) Cherne (4)	Badejo = 21 (18 + 3) (Badejo preto = 2 Badejo queimado = 1)
*23. Rhinobatos* *horkelii*	Viola = 22 (21 + 1) (Viola eletrica = 1)	Viola (11)	Viola (32)	Viola (8) Arraia viola (5)	Cação = 11 (1 + 10) (Cação viola = 10) Viola (6) Arraia viola or Raia viola (6)	Viola (14) Cação = 11 (1 + 10) (Cação viola = 10) Arraia viola or Raia viola (11)	Viola = 23(22 + 1) (Viola macho = 1)
*24. Seriola* *lalandi*	Enchova (13) Olhete (4)	Enchova (4) Olhete (3)	Enchova (17) Olhete (7) Guaraiuba (4) Olho de boi (4) Dnk (4)	Olhete (10)	Olhete (16) Dnk (6)	Olhete (26) Dnk (6)	Olhete (22)
*25. Euthynnus* *alleteratus*	Bonito (22)	Bonito (12)	Bonito (34)	Bonito = 6(3 + 3) (Bonito albacora = 1 Bonito pintado = 1 Bonito serra = 1) Albacora (2)	Bonito (10) Albacora (5) Dnk (6)	Bonito = 16(13 + 3) (Bonito albacora = 1 Bonito pintado = 1 Bonito serra = 1) Albacora (7) Atum (3) Dnk (6)	Cavala = 15 (14 + 1) (Cavala cachorro = 1) Bonito = 8 (7 + 1) (Bonito cachorro = 1) Galhado (4) Cavalinha (4)
*26. Menticirrhus* *americanus*	Papa-terra (19)	Papa-terra (5) Dnk (3)	Papa-terra (24) Dnk (3)	Papa-terra (10)	Papa-terra (20)	Papa-terra (30) Perna de moça (3)	Papa-terra = 23 (21 + 2) (Papa terra preta = 2) Furacu (3)
*27. Dasyatis* *guttata*	Arraia (21)	Arraia = 12 (9 + 3) (Arraia branca = 2 Arraia preta = 1)	Arraia = 33 (30 + 3) (Arraia branca = 2 Arraia preta = 1)	Arraia = 12 (4 + 8) (Arraia mantega = 8)	Arraia or Raia = 23 (3 + 20) Arraia mantega or Raia mantega = 15 Arraia prego = 2 Arraia comum = 1 Arraia rengo = 1 Arraia-morcego = 1)	Arraia or Raia = 35 (7 + 28) Arraia mantega or Raia mantega = 23 Arraia prego = 2 Arraia comum = 1 Arraia rengo = 1 Arraia-morcego = 1)	Arraia = 26 (6 + 20) (Arraia manteiga = 14 Arraia amarela =5 Arraia lixa = 1)
*28. Gymnothorax* *ocellatus*	Mututuca = 9 (8 + 1) (Mututuca pintada = 1) Caramuru or Camburu (5) Dnk (5)	Caramuru or Camburu =6(5 + 1) (Caramburu miroro = 1) Dnk (3)	Mututuca = 10 (9 + 1) (Mututuca pintada = 1) Caramuru or Camburu =11 (10 + 1) (Caramburu miroro = 1) Moreia (3) Dnk (8)	Moreia = 10 (6 + 4) (Moreia fogo = 4)	Moreia = 10 (9 + 1) (Moreia pintada = 1) Dnk (11)	Moreia = 20 (15 + 5) (Moreia fogo = 4 Moreia pintada = 1) Dnk (11)	Moreia = 11 (8 + 3) (Moréia pintada = 1 Moréia venenosa = 1 Moréia amarela = 1) Cobra = 6 (4 + 2) (Cobra do mar = 2) Dnk (4)
*29. Trichiurus* *lepturus*	Espada (20)	Espada (11)	Espada (31)	Espada (10)	Espada (17) Dnk (5)	Espada (27) Dnk (5)	Espada (20)
*30. Pseudoplatystoma* *fasciatum (control)*	Surubim (7) Agua doce (4) Dnk (4)	Surubim (3) Dnk (3)	Surubim (10) Agua doce (4) Dnk (7)	Agua doce (5)	Agua doce (9) Dnk (7)	Agua doce (14) Bagre (3) Dnk (7)	Pintado (5) Dnk (12)
*31. Mycteroperca* *bonaci*	Pirambeba (9) Badejo (6)	Badejo = 3 (2 + 1) (Badejo areia = 1) Piragica (2) Dnk (5)	Pirambeba (10) Badejo = 9 (8 + 1) (Badejo areia = 1) Garoupa (3) Dnk (6)	Badejo = 8 (7 + 1) (Badejo sapão = 1)	Badejo = 14 (10 + 4) (Badejo de areia = 2 Badejo quadrado = 1 Badejo-mira = 1) Garoupa (5) Cherne (3)	Badejo = 22 (17 + 5) (Badejo de areia = 2 Badejo quadrado = 1 Badejo sapão = 1 Badejo-mira = 1) Garoupa (6) Cherne (5) Saltão or Sultão (4)	Badejo = 18 (13 + 5) (Badejo branco = 3 Badejo preto = 1 Badejo queimado = 1) Garoupa (3) Badejo branco (3)
*32. Epinephelus* *marginatus*	Garoupa (4) Badejo (4) Pirambeba (4)	Badejo = 3 (2 + 1) (Badejo preto = 1) Garoupa (2) Dnk (5)	Badejo = 7 (6 + 1) (Badejo preto = 1) Garoupa (6) Pirambeba (5) Dnk (7)	Garoupa (9)	Garoupa = 18 (17 + 1) (Garoupa são tome = 1) Cherne (3)	Garoupa = 27 (26 + 1) (Garoupa são tome = 1) Cherne (5)	Garoupa (22) Garoupeta or Garopeta or Garupeta (5)
*33. Mugil* *curema*	Tainha (22)	Tainha (12)	Tainha (34)	Parati (12)	Parati (14) Tainha (9)	Parati (26) Tainha (10)	Tainha (15) Parati (6) Cara amarela (3) Tanhota or Tainhota (3)
*34. Lutjanus* *cyanopterus*	Caranha = 21 (20 + 1) (Caranha vermeha = 1)	Caranha (11)	Caranha = 32 (31 + 1) (Caranha vermeha = 1)	Vermelho = 6 (1 + 5) (Vermelho caranho =4 Vermelho cioba = 1) Caranha (3)	Vermelho = 12 (1 + 11) (Vermelho caranho(a) = 8 Vermelho dentão = 3) Caranha (6) Dnk (5)	Vermelho = 18 (2 + 16) (Vermelho caranho(a) = 12 Vermelho dentão = 3 Vermelho cioba = 1) Caranha (9) Dnk (5)	Caranha (14) Pescada = 5 (3 + 2) (Pescada amarela = 2)
*35. Caranx* *latus*	Gracaim (9) Xareu (8) Cabecudo (7)	Gracaim (5) Cabecudo (3)	Gracaim (14) Xareu (10) Cabecudo (10)	Faqueco (8)	Faqueco(a) (9) Xerelete = 11(9 + 2) (Xerelete rombudo = 2) Dnk (4)	Faqueco(a) (17) Xerelete = 11(9 + 2) (Xerelete rombudo = 2) Dnk (4)	Xaréu (13) Xerelete (4)
*36. Scomberomorus* *brasiliensis*	Cavala (12) Sororoca (5) Dnk (3)	Cavala (5) Sororoca (2) Dnk (5)	Cavala (17) Sororoca (7) Dnk (8)	Sororoca (8) Cavala (3) Sarda (3)	Cavala (10) Sororoca (10) Sarda (4)	Sororoca (18) Cavala (13) Sarda (7)	Sororoca (20) Cavala (6)
*37. Aluterus* *monoceros*	Peixe rato (14) Peixe folha (8)	Peixe folha (10) Peixe rato (3)	Peixe folha (18) Peixe rato (17)	Chinelo (11)	Perua (9) Cangulo (8) Dnk (3)	Chinelo (11) Perua (9) Cangulo (9)	Peixe porco = 19 (12 + 7) (Peixe porco branco = 5 Peixe porco grande = 2) Cangulu (3)
*38. Aluterus* *schoepfii*	Peixe rato (12) Peixe folha (7) Capado = 4 (Capado branco = 2 Capado folha = 1 Capado rato = 1)	Peixe folha (4) Peixe rato (3) Dnk (3)	Peixe rato (15) Peixe folha (11) Capado = 6 (Capado branco = 4 Capado folha = 1 Capado rato = 1) Dnk (4)	Porco (6) Chinelo (6)	Porco (13) Cangulo = 6 (5 + 1) (Cangulo vassoura = 1)	Porco (19) Chinelo (6) Cangulo = 6 (5 + 1) (Cangulo vassoura = 1)	Peixe porco = 17 (5 + 12) (Peixe porco preto = 8 Peixe porco da pedra = 3 Peixe porco tandé = 1) Porco (3) Cangulu (3)

The numbers in parenthesis refer to generic name plus binomials (example: Sargento includes eight generic plus three binomials). *Dnk* does not know

## Results

The results of the study are here in the same sequence used to show the pictures to the fishers (1 to 38). We interviewed a total of 92 fishers in the three study areas in the coast of Brazil: **NE**, **SE** and **S**. Our results indicate similarities in the fishers’ identification of the generic ranking and the groups of fish formed by the fishers.

### Fish identification and biological information according to fishers’ knowledge

1. *Abudefduf saxatilis* (Linnaeus, 1758)(sargeant-major)
  *Abudefduf saxatilis* is primarily called capiacaba in the northeast (NE) and sargento (generic) and sargento mouro (specific) in the southeast Salgo or sargo is the name used in the south (Table 1). The identification and naming of this species was reasonably homogeneous, and most fishers identified it (Table 1). All fishers said that the habitats of this species are reefs, rocks or coastal areas (Table 3). The diet of this species was also identified, by all respondents, as slime (limo), ooze, detritus (sludge, lodo), and mollusks (marisco). Sludge was cited in the NE, crustaceans and mollusks in the SE (marisco and camarão) and algae (slime, limo) in the S (algae and limo) (Tables 2 and 3).

**Table 2:** The folk diet of the fish shown to 92 fishermen of the coast of Brazil

Species	Porto Sauípe BAHIA (*n* = 22)	Itacimirim BAHIA (*n* = 12)	NORTHEAST: Itacimirim & Porto Sauipe (*n* = 34)	Itaipu NITEROI (*n* = 12)	Copacabana RIO DE JANEIRO RJ (*n* = 23)	SOUTHEAST: Copacabana& Itaipu (*n* = 35)	SOUTHERN: Florianópolis (*n* = 23)
*1. Abudefduf* *saxatilis*	Sludge (11) Shrimp (8) Fish = 5 (sardinha = 1)	Seaweed (4) Shrimp (4) Dnk (4)	Shrimp (11) Sludge (11) Seaweed (6) Fish = 6 (sardinha = 1) Slime (3) Stone (3) Dnk (4)	Shellfish (5) Slime (4)	Fish = 6 (sardinha = 2, manjuba = 1) Shellfish (5) Shrimp (3) Crustacean (3)	Shellfish (10) Fish = 8 (sardinha = 2, manjuba = 2) Crustacean (4) Slime (4)	Slime (10) Alga (6) Shellfish (5) Mole crab (4) Fish = 3 (manjuva = 3)
*2. Bothus* *ocellatus*	Shrimp (11) Mud (5) Fish = 5 (sardinha = 1, pititinga = 1, tainha = 1) Soft crab (3)	Fish = 5 (tainha = 1) Mud (4) Shrimp (3)	Shrimp (14) Fish = 10 (tainha = 2, sardinha = 1, pititinga = 1) Mud (9) Shellfish (3) Soft crab (3)	Fish = 4 (sardinha = 2, manjuba = 1) Shrimp (3) Mud (3) Net (3)	Shrimp (14) Fish = 13 (sardinha = 6, manjuba = 4) Bait (3)	Shrimp (17) Fish = 17 (sardinha = 8, manjuba = 5) Squid (5) Bait (4)	Fish = 17 (manjuva = 15) Shrimp (7) Squid (3)
*3. Stegastes* *leucostictus*	Shrimp (6) Sludge (4) Fish (3) Dnk (8)	Shrimp (3) Dnk (4)	Shrimp (9) Fish = 5 (sardinha = 1) Sludge (4) Alga (3) Dnk (12)	Squid (1) Mussel (1)	Shrimp (3)	Shrimp (3)	Slime (4) Dnk (12)
*4. Canthidermis* *sufflamen*	Fish = 18 (sardinha = 6, avoador = 1, manjuba = 1) Shrimp (6) Anything (4)	Fish = 5 (sardinha = 1) Shrimp (3) Anything (3)	Fish = 23 (sardinha = 7, avoador = 1, manjuba = 1) Shrimp (9) Anything (7) Dnk (3)	Fish = 8 (sardinha = 4, bonito = 1, manjuba = 1) Squid (6) Anything (4)	Shrimp (5) Fish = 5 (sardinha = 3) Squid (4) Anything (6)	Fish = 13 (sardinha = 7, bonito = 1, manjuba = 1) Squid (10) Shrimp (6) Anything (10)	Everything (12) Fish = 9 (manjuva = 4, sardinha = 1) Squid (6) Slime (3)
*5. Pomatomus* *saltatrix*	Fish = 15 (sardinha = 5, manjuba = 1) Shrimp (10) Dnk (4)	Fish = 10 (sardinha = 3) Shrimp (7) Dnk (2)	Fish = 25 (sardinha = 8, manjuba = 1) Shrimp (17) Squid (4) Dnk (6)	Fish = 13 (sardinha = 8, bonito = 2, manjuba = 2, parati = 1) Squid (7)	Fish = 25 (sardinha = 16, manjuba = 5) Squid (5)	Fish = 38 (sardinha = 24, manjuba = 7, bonito = 2, parati = 1) Squid (12)	Fish = 22 (manjuva = 18, espada = 1, sardinha = 1) Squid (6)
*6. Bodianus* *pulchellus*	Fish = 15 (sardinha = 3) Shrimp (8) Sludge (3) Stone (3)	Shrimp (5) Fish = 4 (sardinha = 2)	Fish = 19 (sardinha = 5) Shrimp (13)	Fish = 2 (sardinha = 1 bonito = 1)	Fish = 3 (sardinha = 1 manjubinha = 1) Shrimp (2) Alga (2)	Fish = 5 (sardinha = 2 bonito = 1 manjubinha = 1)	Fish = 7 (manjuva = 5, sardinha = 1) Dnk (12)
*7. Lutjanus* *synagris*	Fish = 18 (sardinha = 9, manjuba = 1 tainha = 1, xinxarro = 1) Shrimp (15)	Fish = 11 (sardinha = 5, xinxarro = 1) Shrimp (6)	Fish = 29 (sardinha = 14, xinxarro = 2, barana = 1, manjuba = 1, tainha = 1) Shrimp (21) Anything (3)	Fish = 7 (sardinha = 4, manjuba = 1) Squid (3)	Shrimp (12) Fish = 9 (sardinha = 6, manjuba = 2) Squid (6)	Fish = 16 (sardinha = 10, manjuba = 3) Shrimp (13) Squid (9)	Fish = 14 (manjuva = 7 sardinha = 1, parati = 1, tanhota = 1) Squid (8) Shrimp (6) Mole crab (6) Earthworm (4)
*8. Centropomus* *parallelus*	Fish = 23 (tainha = 11, sardinha = 4, piaba = 3, pititinga = 1) Shrimp (18)	Fish = 13 (tainha = 4, piaba = 4, pititinga = 1) Shrimp (8)	Fish = 36 (tainha = 15, piaba = 7, sardinha = 4, pititinga = 2) Shrimp (26)	Shrimp (10) Fish = 4 (sardinha = 2, manjuba = 1)	Shrimp (20) Fish = 7 (sardinha = 3, manjuba = 2)	Shrimp (30) Fish = 11 (sardinha = 5, manjuba = 3) Squid (4)	Fish = 21 (manjuva = 20) Shrimp (6)
*9. Bothus* *robinsi*	Shrimp (12) Fish = 8 (tainha = 2, sardinha = 1, piaba = 1) Mud (5)	Fish = 6 (tainha = 1, sardinha = 1, pititinga = 1) Mud (4)	Shrimp (14) Fish = 14 (tainha = 3, sardinha = 2, piaba = 1, pititinga = 1) Mud (9)	Shrimp (4) Fish = 3 (sardinha = 2, manjuba = 1) Net (3)	Shrimp (13) Fish = 13 (sardinha = 6, manjuba = 4) Mole crab (3)	Shrimp (17) Fish = 16 (sardinha = 8, manjuba = 5) Mole crab (4)	Fish = 21 (manjuva = 18) Shrimp (8) Squid (4) Mole crab (3)
*10. Umbrina* *coroides*	Shrimp (19) Fish = 18 (sardinha = 7, tainha = 5)	Shrimp (11) Fish = 4 (sardinha = 3) Mud (3)	Shrimp (30) Fish = 22 (sardinha = 10, tainha = 5)	Mole crab (4) Squid (4) Fish = 4 (sardinha = 2, manjuba = 2)	Mole crab (7) Shrimp (5) Fish = 5 (sardinha = 3) Squid (3)	Mole crab (11) Fish = 9 (sardinha = 5, manjuba = 2) Shrimp (7) Squid (7)	Shrimp (11) Fish = 9 (manjuva = 5) Earthworm (5) Mole crab (4) Squid (3)
*11. Micropogonias* *furnieri*	Shrimp (21) Fish = 19 (tainha = 7, sardinha = 5, avoador = 1)	Shrimp (10) Fish = 7 (sardinha = 4, pititinga = 1) Mud (4)	Shrimp (31) Fish = 26 (sardinha = 9, tainha = 7, avoador = 1, pititinga = 1) Mud (5)	Squid (9) Shrimp (5) Mole crab (3) Fish = 3 (sardinha = 2, manjuba = 1)	Fish = 14 (sardinha = 10, manjuba = 2) Squid (11) Shrimp (8) Mole crab (4)	Squid (20) Fish = 17 (sardinha = 12, manjuba = 3) Shrimp (13) Mole crab (7)	Shrimp (12) Earthworm (8) Squid (7) Fish = 7 (manjuva = 6)
*12. Centropomus* *undecimalis*	Fish = 22 (tainha = 11, sardinha = 3, piaba = 2, pititinga = 1) Shrimp (20)	Fish = 15 (piaba = 5, tainha = 4, sardinha = 2) Shrimp (7)	Fish = 37 (tainha = 15, piaba = 7, sardinha = 5, pititinga = 1) Shrimp (27)	Shrimp (8) Fish = 4 (sardinha = 2, manjuba = 1)	Shrimp (20) Fish = 6 (sardinha = 4, manjuba = 1)	Shrimp (29) Fish = 10 (sardinha = 6, manjuba = 2)	Fish = 17 (manjuva = 17) Shrimp (7) Dnk (3)
*13. Cynoscion* *jamaicensis*	Shrimp (15) Fish = 13 (sardinha = 5, tainha = 3) Dnk (5)	Shrimp (5) Fish = 5 (sardinha = 2, pititinga = 1) Dnk (5)	Shrimp (20) Fish = 18 (sardinha = 7, tainha = 3, pititinga = 1) Dnk (10)	Squid (7) Fish = 5 (sardinha = 3, manjuba = 2) Shrimp (4)	Fish = 11 (sardinha = 6, manjuba = 3) Shrimp (10) Squid (4)	Fish = 16 (sardinha = 9, manjuba = 5) Shrimp (14) Squid (11)	Fish = 21 (manjuva = 19 sardinha = 1) Squid (7) Shrimp (6)
*14. Caranx* *crysos*	Fish = 20 (sardinha = 8, manjuba = 4, tainha = 3, avoador = 1, pititinga = 1) Shrimp (19) Squid (9)	Shrimp (10) Squid (5)	Shrimp (29) Fish = 22 (sardinha = 9, manjuba = 4, tainha = 3, avoador = 1, pititinga = 1) Squid (14)	Squid (10) Fish = 7 (sardinha = 6)	Fish = 19 (sardinha = 11, manjuba = 7) Shrimp (4) Squid (3)	Fish = 26 (sardinha = 17, manjuba = 7) Squid (13) Shrimp (6)	Fish = 20 (manjuva = 18 sardinha = 1) Shrimp (3)
*15. Rhinobatos* *percellens*	Shrimp (19) Fish = 12 (sardinha = 5, tainha = 2)	Shrimp (8) Fish = 6 (sardinha = 3)	Shrimp (27) Fish = 18 (sardinha = 8, tainha = 2)	Squid (9) Fish = 8 (sardinha = 6, bonito = 1) Net (3)	Shrimp (8) Fish = 8 (sardinha = 4, manjuba = 1) Net (4) Mole crab (3)	Fish = 16 (sardinha = 10, manjuba = 1, bonito = 1) Shrimp (9) Squid (9) Net (7)	Shrimp (9) Fish = 9 (manjuva = 4, pescadinha = 1, peixe galego = 1) Earthworm (5) Squid (4) Everything (3)
*16. Oligoplites* *saliens*	Shrimp (20) Fish = 17 (sardinha = 6, manjuba = 2, tainha = 1) Squid (4)	Fish = 11 (pititinga = 3, manjuba = 2, sardinha = 2) Shrimp (7) Squid (3)	Fish = 28 (sardinha = 8, manjuba = 4, pititinga = 3, tainha = 1) Shrimp (27) Squid (7)	Squid (6) Net (6) Fish = 6 (sardinha = 3, manjuba = 3)	Fish = 19 (sardinha = 12, manjuba = 6) Shrimp (4)	Fish = 25 (sardinha = 15, manjuba = 9) Squid (6) Net (6) Shrimp (5)	Fish = 21 (manjuva = 21)
*17. Conger* *orbignianus*	Fish = 15 (sardinha = 1) Shrimp (5) Anything (6) Dnk (3)	Fish = 8 (sardinha = 2, xinxarro = 1) Octopus (4) Anything (3) Dnk (2)	Fish = 23 (sardinha = 3, xinxarro = 1) Shrimp (6) Octopus (4) Anything (9) Dnk (5)	Fish = 9 (sardinha = 5, bonito = 2, espada = 1) Squid (6) Anything (5)	Fish = 9 (sardinha = 4, manjuba = 1) Anything (5)	Fish = 18 (sardinha = 9, bonito = 2, espada = 1, manjuba = 1) Squid (8) Anything (10)	Fish = 6 (manjuva = 3) Shrimp (3) Everything (3) Dnk (4)
*18. Sphoeroides* *dorsalis*	Shrimp (11) Fish = 11 (tainha = 2, sardinha = 1) Oyster (3) Anything (6)	Shrimp (5) Anything (4) Dnk (3)	Shrimp (16) Fish = 13 (tainha = 2, sardinha = 2) Anything (10) Dnk (4)	Squid (7) Fish = 5 (sardinha = 4) Anything (4)	Fish = 11 (sardinha = 6, baiacu = 1) Shrimp (3) Squid (3) All (3) Anything (5)	Fish = 16 (sardinha = 10, baiacu = 1) Squid (10) Shrimp (4) Anything (9)	Everything (10) Fish = 7 (manjuva = 4, sardinha = 1) Shrimp (3)
*19.Bodianus* *rufus*	Fish = 12 (sardinha = 3) Shrimp (10) Sludge (5)	Shrimp (3) Сrab (3)	Fish = 14 (sardinha = 5) Shrimp (13) Sludge (5)	Shellfish (1) Fish = 1 (sardinha = 1) Coral (1) Squid (1)	Shrimp (2) Shellfish (2) Dnk (3)	Shellfish (3) Dnk (3)	Fish = 3 (manjuva = 2) Dnk (15)
*20. Gymnothorax* *funebris*	Fish (13) Octopus (3) Anything (6) Dnk (3)	Fish = 11 (sardinha = 1, voador = 1, xinxarro = 1) Octopus (4) Anything (3)	Fish = 24 (sardinha = 1, voador = 1, xinxarro = 1) Octopus (7) Anything (9)	Fish = 7 (sardinha = 5, cavala = 1) Anything (4)	Fish = 9 (sardinha = 3) Anything (7)	Fish = 16 (sardinha = 8, cavala = 1) Anything (11)	Fish = 4 (manjuva = 1, anchova = 1) Squid (3) Dnk (9)
*21. Sphoeroides* *spengleri*	Shrimp (11) Fish = 7 (sardinha = 2) Oyster (5) Сrab (3) Anything (8)	Shrimp (5) Anything (6)	Shrimp (16) Fish = 9 (sardinha = 2) Oyster (5) Anything (14)	Squid (6) Fish = 6 (sardinha = 5) Anything (5)	Fish = 15 (sardinha = 8, baiacu = 1, manjuba = 1) Squid (3) Anything (6)	Fish = 21 (sardinha = 13, baiacu = 1, manjuba = 1) Squid (9) Anything (11)	Everything (10) Fish = 8 (manjuva = 4)
*22. Mycteroperca* *acutirostris*	Fish = 24 (sardinha = 5, avoador = 1, guaricema = 1, tainha = 1, xinxarro = 1)	Fish = 13 (xinxarro = 2, sardinha = 1, guaricema = 1)	Fish = 37 (sardinha = 6, xinxarro = 3, guaricema = 2, avoador = 1, tainha = 1)	Shrimp (8) Squid (5) Fish = 5 (sardinha = 4, bonito = 1)	Shrimp (19) Fish = 9 (sardinha = 4, parati = 1, manjuba = 1) Squid (4)	Shrimp (27) Fish = 14 (sardinha = 8, parati = 1, manjuba = 1, bonito = 1) Squid (9)	Fish = 19 (manjuva = 19) Shrimp (12)
*23. Rhinobatos* *horkelii*	Shrimp (17) Fish = 13 (sardinha = 6, tainha = 3) Barata (3) Dnk (3)	Shrimp (8) Fish = 6 (sardinha = 3)	Shrimp (25) Fish = 19 (sardinha = 9, tainha = 3) Dnk (4)	Fish = 6 (sardinha = 4, bonito = 1) Net (5) Squid (4)	Shrimp (9) Fish = 8 (sardinha = 3, parati = 1, manjuba = 1) Squid (3) Net (3)	Fish = 14 (sardinha = 7, parati = 1, manjuba = 1, bonito = 1) Shrimp (10) Net (8) Squid (7)	Shrimp (10) Fish = 7 (manjuva = 3, peixe rasteiro = 1, peixe galego = 1) Squid (4) Earthworm (4) Everything (3)
*24. Seriola* *lalandi*	Fish = 24 (sardinha = 11, xinxarro = 2, manjuba = 2, avoador = 1, guaricema = 1, tainha = 1) Squid (6) Shrimp (5)	Fish = 10 (sardinha = 3, guaricema = 2, xinxarro = 2) Shrimp (5)	Fish = 34 (sardinha = 14, xinxarro = 4, guaricema = 3, manjuba = 2, avoador = 1, tainha = 1) Shrimp (10) Squid (7) Dnk (4)	Squid (11) Fish = 5 (sardinha = 5)	Fish = 15 (sardinha = 10, manjuba = 2, cocoroca = 1) Squid (8) Shrimp (4)	Fish = 20 (sardinha = 15, manjuba = 2, cocoroca = 1) Squid (19) Shrimp (5)	Fish = 15 (manjuva = 13, sardinha = 2) Squid (12)
*25. Euthynnus* *alleteratus*	Fish = 25 (sardinha = 14, manjuba = 2, pititinga = 1, tainha = 1, avoador = 1) Shrimp (7) Squid (6)	Fish = 17 (sardinha = 7, manjuba = 1, pititinga = 1, voador = 1, xinxarro = 1)	Fish = 42 (sardinha = 21, manjuba = 3, pititinga = 2, tainha = 1, avoador = 1, voador = 1) Shrimp (9) Squid (8)	Fish = 5 (sardinha = 4, manjuba = 1)	Fish = 12 (sardinha = 9, manjuba = 2)	Fish = 17 (sardinha = 13, manjuba = 3)	Fish = 21 (manjuva = 21)
*26. Menticirrhus* *americanus*	Shrimp (19) Fish = 15 (sardinha = 5, tainha = 3, manjuba = 1) Barata (4)	Shrimp (11) Fish = 6 (sardinha = 4)	Shrimp (30) Fish = 21 (sardinha = 9, tainha = 3, manjuba = 1)	Squid (6) Fish = 5 (sardinha = 5) Shrimp (4)	Shrimp (9) Mole crab (9) Fish = 8 (sardinha = 6, manjuba = 1) Squid (4) Shellfish (4)	Shrimp (13) Fish = 13 (sardinha = 11, manjuba = 1) Mole crab (11) Squid (10) Shellfish (4)	Fish = 12 (manjuva = 8) Mole crab (8) Shrimp (7) Earthworm (5) Squid (5)
*27. Dasyatis* *guttata*	Fish = 19 (sardinha = 3, tainha = 1, barbudo = 1) Shrimp (8) Soft crab (3)	Fish = 9 (sardinha = 1, xinxarro = 1, mutuca = 1) Shrimp (5)	Fish = 28 (sardinha = 4, tainha = 1, xinxarro = 1, mutuca = 1, barbudo = 1) Shrimp (13)	Squid (6) Fish = 6 (sardinha = 4, bonito = 1, manjuba = 1) Net (3)	Fish = 13 (sardinha = 8, manjuba = 2) Squid (5) Anything (3)	Fish = 19 (sardinha = 12, manjuba = 3, bonito = 1) Squid (11) Net (5)	Shrimp (9) Fish = 9 (manjuva = 6) Squid (4) Earthworm (3) Everything (3)
*28. Gymnothorax* *ocellatus*	Fish = 10 (sardinha = 1, tainha = 1) Shrimp (5) Soft crab (3) Anything (3) Dnk (5)	Fish (5) Dnk (3)	Fish = 15 (sardinha = 1, tainha = 1) Shrimp (7) Anything (5) Dnk (8)	Fish = 9 (sardinha = 7, cavala = 1 bonito = 1) Squid (5) Anything (4)	Fish = 4 (sardinha = 4)	Fish = 13 (sardinha = 11, cavala = 1 bonito = 1) Squid (6) Anything (5)	Fish = 11 (manjuva = 6) Squid (6) Bait (4) Soft crab (3) Shrimp (3) Dnk (4)
*29. Trichiurus* *lepturus*	Fish = 23 (sardinha = 6, tainha = 3, manjuba = 2) Shrimp (10)	Fish = 11 (sardinha = 2, pititinga = 2) Shrimp (3)	Fish = 34 (sardinha = 8, tainha = 3, manjuba = 2, pititinga = 2) Shrimp (13)	Fish = 11 (sardinha = 7, manjuba = 2, espada = 1) Squid (4)	Fish = 17 (sardinha = 14, manjuba = 2) Anything (3)	Fish = 28 (sardinha = 21, manjuba = 4, espada = 1) Squid (6) Anything (4)	Fish = 21 (manjuva = 17, sardinha = 3) Squid (5)
*30. Pseudoplatystoma* *fasciatum (control)*	Dnk (17)	Dnk (9)	Dnk (26)	Fish = 1 (sardinha = 1) Squid (1)	Fish (2) Bait (1)	Fish = 3 (sardinha = 1) Squid (1) Bait (1)	Dnk (17)
*31. Mycteroperca* *bonaci*	Fish = 18 (xinxarro = 1, avoador = 1, guaricema = 1, sardinha = 1) Shrimp (3)	Fish = 10 (xinxarro = 1, guaricema = 1, sardinha = 1) Dnk (3)	Fish = 28 (xinxarro = 2, sardinha = 2, guaricema = 2, avoador = 1) Shrimp (4) Dnk (4)	Squid (8) Fish = 8 (sardinha = 7, bonito = 1) Shrimp (5)	Shrimp (13) Fish = 12 (sardinha = 8, manjuba = 1, cavalinha = 1) Squid (3)	Fish = 20 (sardinha = 15, bonito = 1, manjuba = 1, cavalinha = 1) Shrimp (18) Squid (11)	Fish = 21 (manjuva = 16, bonito = 2, cavalinha = 1, sardinha = 1) Shrimp (8)
*32. Epinephelus* *marginatus*	Fish = 21 (saramonete = 1, sardinha = 1, vermelho = 1, xinxarro = 1, caramburu = 1) Dnk (3)	Fish = 8 (sardinha = 1, xinxarro = 1, guaricema = 1)	Fish = 29 (xinxarro = 2, sardinha = 2, caramburu = 1, guaricema = 1, saramonete = 1, vermelho = 1) Dnk (5)	Fish = 12 (sardinha = 7, bonito = 3, cavala = 2) Squid (5)	Fish = 13 (sardinha = 9, cavalinha = 1) Crustacean (4) Squid (4) Shrimp (3) Mergulho (3)	Fish = 25 (sardinha = 16, bonito = 3, cavala = 2 cavalinha = 1) Squid (9) Crustacean (5)	Fish = 30 (sardinha = 8, bonito = 7, manjuva = 7, anchova = 2, cavalinha = 2, tanhota = 1) Squid (8) Everything (3)
*33. Mugil* *curema*	Mud (22)	Mud (6) Slime (3)	Mud (28)	Net (10)	Bread crumbs (5) Fish = 5 (sardinha = 3, manjuba = 2) Slime (3)	Net (10) Bread crumbs (6) Fish = 5 (sardinha = 3, manjuba = 2) Slime (4)	Areia (12) Mud (5) Slime (3)
*34. Lutjanus* *cyanopterus*	Fish = 24 (tainha = 6, sardinha = 4, avoador = 1) Soft crab (5) Shrimp (4)	Fish = 13 (tainha = 3, xinxarro = 2, bonito = 1, carapitanga = 1, guaricema = 1, voador = 1) Anything (3)	Fish = 37 (tainha = 9, sardinha = 4, xinxarro = 2, bonito = 1, carapitanga = 1, guaricema = 1, avoador = 1, voador = 1) Shrimp (5) Soft crab (5) Anything (4)	Fish = 10 (sardinha = 7, bonito = 2, cavala = 1) Squid (3)	Fish = 12 (sardinha = 6, manjuba = 2, cocoroca = 1) Shrimp (8)	Fish = 22 (sardinha = 13, bonito = 2, manjuba = 2, cocoroca = 1 cavala = 1) Shrimp (10) Squid (5)	Fish = 14 (manjuva = 9, bonito = 1, sardinha = 1) Everything (4) Squid (3) Shrimp (3)
*35. Caranx* *latus*	Fish = 21 (sardinha = 7, manjuba = 3) Shrimp (11) Squid (6)	Fish = 8 (sardinha = 2, xinxarro = 1, manjuba = 1, voador = 1) Shrimp (7) Squid (6)	Fish = 29 (sardinha = 9, manjuba = 4, xinxarro = 1, voador = 1) Shrimp (18) Squid (12)	Squid (9) Net (3) Fish = 3 (sardinha = 3) Bait (3)	Fish = 16 (sardinha = 10, manjuba = 6) Shrimp (5) Squid (4)	Fish = 19 (sardinha = 13, manjuba = 6) Squid (13) Shrimp (7)	Fish = 19 (manjuva = 19)
*36. Scomberomorus* *brasiliensis*	Fish = 27 (sardinha = 10, xinxarro = 4, manjuba = 4, guaricema = 1, voador = 1) Squid (3)	Fish = 9 (sardinha = 2, xinxarro = 2, manjuba = 1, barbudo = 1, pititinga = 1) Squid (3) Dnk (3)	Fish = 36 (sardinha = 12, xinxarro = 6, manjuba = 5, pititinga = 1, guaricema = 1, barbudo = 1, voador = 1) Squid (6) Shrimp (4) Dnk (5)	Net (6) Squid (4) Fish = 4 (sardinha = 3)	Fish = 18 (sardinha = 13, manjuba = 3) Shrimp (6)	Fish = 22 (sardinha = 16, manjuba = 3) Net (8) Shrimp (7) Squid (5)	Fish = 22 (manjuva = 21, sardinha = 1)
*37. Aluterus* *monoceros*	Fish = 11 (sardinha = 3, xinxarro = 1) Shrimp (8) Anything (7)	Fish = 4 (xinxarro = 1, voador = 1) Shrimp (3) Anything (8)	Fish = 15 (sardinha = 3, xinxarro = 2, voador = 1) Shrimp (11) Anything (15)	Squid (9) Net (4) Fish = 4 (sardinha = 4)	Shrimp (9) Squid (7) Fish = 7 (sardinha = 5) Anything (3)	Squid (16) Shrimp (11) Fish = 11 (sardinha = 9) Net (5) Anything (5)	Fish = 12 (manjuva = 10) Everything (8) Squid (3)
*38. Aluterus* *schoepfii*	Fish = 9 (sardinha = 2, xinxarro = 1) Shrimp (5) Anything (7)	Shrimp (3) Anything (3)	Fish = 11 (sardinha = 3, xinxarro = 1) Shrimp (8) Anything (10)	Squid (8) Fish = 5 (sardinha = 5) Anything (3)	Fish = 8 (sardinha = 4) Shrimp (4) Squid (4)	Fish = 13 (sardinha = 9) Squid (12) Shrimp (6) Anything (4)	Fish = 7 (manjuva = 6) Everything (6) Slime (3) Squid (3)

*Dnk* does not know

**Table 3:** The habitat of fish according to the fishermen of the coast of Brazil (five citations or more) (*n* = 92)

Species	Porto Sauípe BAHIA (*n* = 22)	Itacimirim BAHIA (*n* = 12)	Northeast: Itacimirim & Porto Sauipe (*n* = 34)	Itaipu NITEROI RJ (*n* = 12)	Copacabana RIO DE JANEIRO RJ (*n* = 23)	Southeast: Copacabana& Itaipu (*n* = 35)	Southern: Florianópolis (*n* = 23)
*1. Abudefduf* *saxatilis*	Rock (19) Arrecife (3)	Arrecife (5) Rock (4) Dnk (3)	Rock (23) Arrecife (8) Dnk (4)	Rock (11)	Rock (18)	Rock (29)	Rock (21)
*2. Bothus* *ocellatus*	Mud (17) River (13) Sea (9) Sand (3)	Sand (5) Mud (3) Sea (3)	Mud (20) River (15) Sea (12) Sand (8)	Sand (11)	Sand (13) Gravel (5) Rock (3)	Sand (24) Gravel (5) Rock (5)	Bottom (15) Sand (10) Mud (6)
*3. Stegastes* *leucostictus*	Rock (14) Bottom (4) Dnk (5)	Arrecife (5) Rock (4) Dnk (4)	Rock (18) Arrecife (6) Bottom (4) Dnk (9)	Rock (3)	Rock (6)	Rock (9)	Rock (7) Dnk (11)
*4. Canthidermis* *sufflamen*	Rock (13) Water column (3) Meia peca (3)	Rock (9)	Rock (22) Meia peca (5) Water column (3)	Rock (8)	Rock (16) Gravel (3)	Rock (24) Gravel (5) Sand (4)	Water column (11) Bottom (7) Largo (4) Rock (3) Midwater (3)
*5. Pomatomus* *saltatrix*	Water column (8) Mud (7)	Mud (3)	Mud (10) Water column (8) Migratory (4) At large (3) Dnk (4)	Rock (7) At large (5)	Rock (11) Migratory (5) At large (3)	Rock (18) At large (8) Migratory (5)	Reef (9) Water column (9) Bottom (7) High sea (4) Islands (3) Rock (3)
*6. Bodianus* *pulchellus*	Rock (20)	Rock (9) Arrecife (5)	Rock (29) Arrecife (5) At large (4)	Rock (3)	Rock (9)	Rock (12)	Rock (8) Bottom (4) Dnk (10)
*7. Lutjanus* *synagris*	Rock (14) Mud (6)	Rock (9) Mud (3)	Rock (23) Mud (9)	Rock (7)	Rock (11) Sand (3)	Rock (18)	Bottom (11) Rock (6)
*8. Centropomus* *parallelus*	River (17) Sea (17)	River (10) Sea (3)	River (27) Sea (20) Mud (4)	Rock (7) Lagoon (4)	Rock (10) Shoreline & Sea (4) Sand (3) Baía (3) Lagoon (3) Mangrove (3)	Rock (17) Lagoon (7)	Rock (18) Bottom (3)
*9. Bothus* *robinsi*	Mud (15) River (12) Sea (8) Rock (3)	Sand (5) Sea (3) Mud (3) River (3)	Mud (18) River (15) Sea (11) Sand (6) Rock (4)	Sand (9)	Sand (13) Gravel (7)	Sand (22) Gravel (7)	Bottom (14) Sand (10) Mud (6)
*10. Umbrina* *coroides*	Mud (18) Baía (3) Shoreline & Sea (3)	Mud (11) Shoreline (4)	Mud (29) Shoreline & Sea (7)	Sand (5) Shoreline (3)	Shoreline (11) Sand (7)	Shoreline (14) Sand (12)	Bottom (16) Sand (4) Mud (3)
*11. Micropogonias* *furnieri*	Mud (17)	Mud (11) Shoreline (3)	Mud (28) Shoreline & Sea (6)	Sand (5) Mud (4)	Sand (9) Gravel (5) At large (4) Mud (3)	Sand (14) Gravel (7) Mud (7) At large (5)	Bottom (16) Water column (5) Sand (3)
*12. Centropomus* *undecimalis*	River (17) Sea (14) Freshwater (3) Salt water (3)	River (8) Sea (5)	River (25) Sea (19) Freshwater (3) Salt water (3)	Rock (6) Lagoon (4)	Rock (9) Lagoon (4) Sea (3) Baía (3)	Rock (15) Lagoon (8)	Rock (15) Bottom (4) Water column (3)
*13. Cynoscion* *jamaicensis*	Mud (11) Baía (3) Dnk (5)	Mud (6) Dnk (5)	Mud (17) Dnk (10)	Sand (7)	Sand (6) At large (4)	Sand (13) At large (5)	Bottom (16) Sand (5) Water column (3)
*14. Caranx* *crysos*	Water column (8) Sea (4) Rock (3)	Rock (6) Meia peca (3)	Water column (10) Rock (9) Meia peca (5) Mud (4) Sea (4)	Rock (7) Sand (4)	Rock (11) Migratory (4) Shoreline (3) At large (3)	Rock (18) Migratory (6) At large (5) Sand (4)	Water column (13) Bottom (4) High sea (3) Sand (3)
*15. Rhinobatos* *percellens*	Mud (16) Sand (3)	Mud (9)	Mud (25) Sand (5) Rock (3)	Gravel (6) At large (3)	Sand (15) Bottom (5) Gravel (5) At large (5)	Sand (17) Gravel (11) At large (8) Bottom (5)	Bottom (19) Sand (6)
*16. Oligoplites* *saliens*	Water column (6) Mud (5) Rock (4) Any place (3)	Mud (6)	Mud (11) Water column (6) Rock (5) Any place (4)	Migratory (6) Sand (3)	Sand (4) At large (4) Baía (3)	Migratory (8) Sand (7) At large (5)	Water column (13) Bottom (4)
*17. Conger* *orbignianus*	Rock (13) Mud (6)	Rock (6)	Rock (19) Mud (7)	Rock (5) Mud (3)	Rock (17) At large (3)	Rock (22) At large (4)	Bottom (9) Rock (7) Mud (4) Dnk (4)
*18. Sphoeroides* *dorsalis*	Sea (8) River (8) Mud (4) Water column (3)	Sand (3) Mud (3)	Sea (8) River (8) Mud (7) Rock (4) Raso (4)	Rock (4) Sand (3)	Sand (5) Rock (4) Shoreline (3)	Sand (8) Rock (8)	Bottom (12) Water column (9) Dnk (4)
*19.Bodianus* *rufus*	Rock (20)	Rock (6) Arrecife (5)	Rock (26) Arrecife (5)	Rock (3)	Rock (10)	Rock (13)	Rock (4) Freshwater (3) Dnk (4)
*20. Gymnothorax* *funebris*	Rock (19) Dnk (3)	Rock (8)	Rock (27)	Rock (9)	Rock (15)	Rock (24)	Rock (9) Reef (3) Dnk (8)
*21. Sphoeroides* *spengleri*	Sea (9) River (8) Mud (3)	Mud (4) Sand (3) Rock (3)	Sea (9) River (8) Mud (7) Rock (4) Any place (4)	Rock (4) Sand (3) Any place (3)	Sand (6) Shoreline (3) Rock (3)	Sand (9) Rock (7) Shoreline (4) Any place (4)	Bottom (12) Water column (9) Midwater (3)
*22. Mycteroperca* *acutirostris*	Rock (20) At large (6)	Rock (9) At large (3)	Rock (29) At large (9)	Rock (10)	Rock (22)	Rock (32)	Rock (26)
*23. Rhinobatos* *horkelii*	Mud (18) Sand (3)	Mud (9)	Mud (27) Sand 5) Rock (4)	At large (4) Sand (3)	Sand (11) Rock (3) Gravel (3)	Sand (14) Gravel (5) At large (5) Rock (4)	Bottom (18) Sand (7)
*24. Seriola* *lalandi*	Water column (8) At large (5) Rock (4)	Rock (5) Meia peca (3)	Rock (9) Water column (8) At large (7) Meia peca (5)	Rock (6) At large (3)	Rock (13) At large (3) Islands (3) Migratory (3)	Rock (19) At large (6) Islands (4)	Rock (10) Water column (8) Islands (5) Bottom (5) High sea (4) Reef (4)
*25. Euthynnus* *alleteratus*	Water column (13) Migratory (6)	Rock (3) Meia peca (3) Any place (3)	Water column (13) Migratory (7) Rock (5) Meia peca (5) Mud (4) Any place (4)	At large (7)	At large (8) Migratory (5)	At large (15) Migratory (5)	Water column (14) High sea (8) Bottom (4)
*26. Menticirrhus* *americanus*	Mud (18) Sand (3) Shoreline (3)	Mud (9) Shoreline (4)	Mud (27) Shoreline (7)	Sand (8) Shoreline (3)	Sand (11) Shoreline (11)	Sand (19) Shoreline (14)	Bottom (14) Sand (5)
*27. Dasyatis* *guttata*	Mud (16) Rock (5) Sea (4)	Mud (7) Rock (3) Any place (4)	Mud (23) Rock (8) Any place (4)	Sand (4)	Sand (12) Bottom (5) Gravel (4) At large (4) Migratory (3)	Sand (16) Gravel (6) At large (6) Bottom (5)	Bottom (17) Sand (5) Mud (3)
*28. Gymnothorax* *ocellatus*	Rock (10) Mud (8) Dnk (4)	Dnk (3)	Rock (12) Mud (10) Dnk (7)	Rock (7)	Rock (7)	Rock (14)	Rock (13) Bottom (5) Reef (3) Dnk (4)
*29. Trichiurus* *lepturus*	Mud (6) Water column (4)	Mud (5)	Mud (11) Water column (4)	Rock (3)	Sand (7) Rock (4)	Sand (9) Rock (7)	Bottom (13) Water column (12) High sea (8) Rock (5)
*30. Pseudoplatystoma* *fasciatum(control)*	Freshwater (4) Dnk (12)	Dnk (9)	Freshwater (5) Dnk (21)	Sand (1)	Freshwater (2)	Freshwater (2)	Freshwater (12) Dnk (9)
*31. Mycteroperca* *bonaci*	Rock (18) At large (5) Bottom (3)	Rock (5) Dnk (3)	Rock (23) At large (6) Dnk (4)	Rock (10)	Rock (19)	Rock (29)	Rock (20)
*32. Epinephelus* *marginatus*	Rock (17) At large (5) Bottom (4) Mud (3)	Rock (9)	Rock (26) At large (6) Bottom (4) Dnk (4)	Rock (10)	Rock (20)	Rock (30)	Rock (22) Reef (7)
*33. Mugil* *curema*	River (20) Sea & Shoreline (14) Mud (6) Water column (3)	River (8) Arrecife (4) Sea & Shoreline (4) Mud (3) Rock (3)	River (28) Sea & Shoreline (18) Mud (9) Arrecife (4)	Migratory (5) Shoreline (3) Lagoon (3) Rock (3)	Sand (7) Lagoon (6) Shoreline (5) Bahia (3)	Sand (9) Lagoon (9) Shoreline (8) Rock (5) Migratory (5)	Water column (15) Bottom (4) Midwater (3) Mud (3) Lagoon (3)
*34. Lutjanus* *cyanopterus*	Rock (18) River (10) Sea (9)	Rock (10) River (3)	Rock (28) River (13) Sea (9)	Rock (8)	Rock (14) Arrecife (3)	Rock (22) Arrecife (4)	Rock (14) Bottom (7) Largo (3) Sand (3)
*35. Caranx* *latus*	Rock (9) Water column (5) Sea (5) Meia peca (3) River (3)	Rock (8)	Rock (17) Water column (6) Sea (6) Meia peca (5)	Rock (8) Shoreline (3)	Rock (6) Sand (3) Shoreline (3)	Rock (14) Shoreline (6) Sand (4)	Water column (8) Bottom (7) Rock (8) High sea (3)
*36. Scomberomorus* *brasiliensis*	Water column (10) Rock (3) Sea (3)	Migratory (3) Shoreline (3) Rock (3) Dnk (3)	Water column (10) Rock (6) Shoreline & Sea (6) Migratory (5) Dnk (4)	Migratory (5) Sand (3)	At large (8) Migratory (7) Sand (3)	Migratory (12) At large (9) Sand (6)	Water column (10) Bottom (6) Rock (3)
*37. Aluterus* *monoceros*	Water column (10) At large (3) Mud (3) Any place (4)	Rock (3) Any place (4)	Water column (11) At large (5) Rock (5) Mud (4) Any place (8)	Rock (7)	Rock (8) At large (3) Gravel (3) Sand (3)	Rock (15) At large (4) Gravel (4) Sand (4)	Rock (12) Bottom (8) Water column (7) High sea (3)
*38. Aluterus* *schoepfii*	Water column (10) At large (3) Mud (3) Any place (3)	Rock (5)	Water column (11) Rock (7) At large (4) Any place (4)	Rock (7)	Rock (7) Sand (4) Gravel (3)	Rock (14) Sand (6) Gravel (4)	Rock (15) Bottom (5) Water column (3)

*Dnk* does not know
2. *Bothus ocellatus* (Agassiz, 1831) (eyed flounder)
  *Bothus ocellatus* (eyed flounder) is called aramaçá by the majority of NE fishers; linguado is the name mostly used in the SE and south of Brazil. Three fishers from the south used binomials (Table 1). Shrimp and fish were mentioned as food by the fishers; mud (ooze) was mentioned in the NE (Table 2). Its habitat was described by most fishers as mud in the NE) (sand was also cited by eight fishers), as sand in the SE, although gravel was also mentioned, and both mud and sand in the S (Table 3).
3. *Stegastes leucostictus* (Müller & Troschel, 1848) (Beaugregory)
  *Stegastes leucostictus* is called pantucano or plantucano in the NE and Ze Pereira (4). However, this species was not recognized by most fishers (Table 1). Its habitat is described as rocky and reefs (Table 3). Despite respondents’ lack of knowledge, the fishers mentioned shrimp as an important food. Ooze and algae were mentioned in the NE (Tables 2 and 3).
4. *Canthidermis sufflamen (*Mitchill, 1815) (Ocean triggerfish)
  *Canthidermis sufflamen* (ocean triggerfish) is called especially Capado branco, and Piracá by NE fishers. In the SE, cangulo and porco are mostly used; in the south, most named it ‘peixe-porco’, with two binomials, peixe-porco branco and peixe-porco legítimo, used (Table 1). Porco is in fact an abbreviation of peixe-porco in the SE. We hear the complete name in both Copacabana and Itaipu.
  Fishers from the NE and SE stated that this is a rocky fish; meia-peça was mentioned by five fishers from the NE. Meia-peça is a name used on the coast of Bahia, where fishers use depth to define fish location and identify habitats. Fishers from Bahia (Porto do Sauípe and Itacimirim) classify habitats as follows (Begossi et al., [27]:109): pedras: 20 fathoms or 30 m deep; ‘baixio’ 27 to 30 or 40–45 m deep; ‘meia-peça’: 34–45 fathoms or 51–67 m deep; and ‘parede’ (literally, “the wall”, meaning the end of the continental shelf): 60/70–90/105 m deep.) Fishers’ reference to the “the wall”, in Bahia State, was already observed in the community of Arembepe [31], as an important fishing area. Small-scale fishers can fish at the end of the continental shelf for it being narrow on the northeastern coast of Brazil.
  The diet of *C. sufflamen* was identified as fish or shrimp by fishers of the NE and SE (21 in the NE and 11 in the SE); sardines were mentioned and are included in the fish category; squid was mentioned in the SE (10) and in the south (6). In this area, most fishers mentioned that this species eats everything (12) (Tables 2 and 3).
5. *Pomatomus saltatrix* (Linnaeus, 1766) (bluefish)
  Enchova (or anchova in the south) is the name given to *P. saltatrix* (Table 1). This species’ habitat is identified as mud or within the water column (NE). This species behaves like a migratory fish, called by fishers “andarilho” (hiker, traveller), and it is also considered migratory in the SE and S, although its habitat there is identified as rocky substrates or reefs. Fish and shrimp are noted as its food in all areas.
6. *Bodianus pulchellus* (Poey, 1860) (spotfin hogfish)
  *Bodianus,* called plantucano or pantucano in the NE, is referred to as budião in the SE (Table 1). Southern fishers did not recognize this species, which is called a rocky fish by fishers from all areas (even in the south where fishers did not recognize it) (Table 1). Fish and shrimp were cited as food (Table 2).
7. *Lutjanus synagris (Linnaeus, 1758)* (lane snapper)
  *Lutjanus synagris* (lane snapper) is called ariocó in the NE (20 of 34 respondents) and vermelho, vermelho cióba or vermelho caranha in the SE and south (Table 1). Fishers associate this species with rocky substrates and primarily mention shrimp and then fish and squid as food items in its diet (Tables 2 and 3)
8. *Centropomus parallelus* (Poey, 1860)(fat snook)
  *Centropomus parallelus* (fat snook) is called robalo in all sites. Binomials occur in the NE, mostly robalo-corcunda and robalo-furão (Table 1). In the SE, this species is called robalo-flecha [32]. Its habitat is identified in the NE as the sea and the river. Rocky substrates are mentioned in the SE and in the south. Primarily shrimp, and then fish, are cited as important food items (Tables 2 and 3).
9. *Bothus robinsi* (Topp & Hoff, 1972) (twospot flounder).
  Two-spot flounder is called aramaçá in the NE and it is called linguado in the SE and S (Table 1), just as *B. ocellatus* (this study, no. 2). This species can also be called solha or soia (Table 1). Linguado da areia, linguado de vaca, and linguado cascalho are some other binomials that were mentioned. Mud, river and sea are cited as habitats of *B. robinsi* in the NE, sand in the SE and rivers and sea bottoms in the south Shrimp and mud are the food cited in the NE and shrimp and fish in the SE and south (Tables 2 and 3).
10. * Umbrina coroides* (Cuvier, 1830) (sand drum)
  The sand drum is primarily called roncador at all sites. Secondarily, it is called riscadinha in the SE and maria luiza in the S. Binomials such as roncador judeus and roncador branco are important (Table 1). Its habitat is cited as mud in the NE and sand or shallow waters in the SE. In the S, both sand and mud are mentioned; however, the bottom is cited in particular. Crustaceans (shrimp, tatui [mole crabs]) are particularly cited, followed by fish (Tables 2 and 3).
11. * Micropogonias furnieri* (Desmarest, 1823) (Whitemouth croaker)
  Whitemouth croaker is called papa-terra and corvina in the NE and corvina in the SE and S (Table 1). Its habitat is noted as mud in the NE, sand in the SE and the sea bottom in the S. Its most important food items are crustaceans (shrimp, mole crab) and fish. Squid was also mentioned (Tables 2 and 3).
12. * Centropomus undecimalis* (Bloch, 1792) (common snook)
  The common snook is essentially called robalo in all areas. Two binomials are of relative importance in the NE: robalo corcunda and robalo cambriacu. Another binomial was mentioned in the SE: robalo flecha (Table 1). Shrimp and fish are the food items mentioned by fishers (Table 2). The literature shows its name as robalo peba [32]. Fishers are aware of the migratory movements of the snook between salt and freshwater (Table 3).
13. * Cynoscion jamaicensis* (Vaillant & Bocourt, 1883) (Jamaica weakfish)
  In the NE, the names samucanga, pescada and perna de moça are mentioned; in the NE, approximately 14 fishers did not recognize this fish. Pescada, pescada-goete, goete and perna de moça were mentioned in the SE. In the south, pescada, pescadinha and pescada (or pescadinha) de bucho are used (Table 1). This species is cited as feeding on shrimp and fish (NE), and in the SE and S fishers included squid as its food (Table 2). Its habitat is identified in particular as mud in the NE, sand in the SE and the bottom in the S (sandy bottom) (Table 3).
14. * Caranx crysos* (Mitchill, 1815) (Blue runner)
  The blue runner was easily recognized, called guaricema in the NE, xerelete in the SE and canarinho, manezinho, or xareu in the S (Table 1). Shrimp, squid and fish were mentioned in all areas as major food items (Table 2). Its habitat was noted as rocky although this species is considered to be located in the water column (‘corrente’, ‘boiado’) and migratory (“andarilho”)(Table 3).
15. * Rhinobatos percellens* (Walbaum, 1792) (Chola guitarfish)
  This species is called viola in the NE and in the S. In the SE, binomials such as arraia viola and cação viola were mentioned (Table 1). Food was identified primarily as shrimp in the NE and fish, shrimp and squid in the SE and S (Table 2). This species is noted as living in a muddy habitat in the NE, in the gravel or sandy bottom in the SE, and on the sea bottom in the S (sandy) (Table 3)
16. * Oligoplites saliens* (Bloch, 1793) (castin leatherjacket)
  Called guaibira or guaivira in all sites, this species is also called solteira in the NE. This fish was quite well recognized (Table 1). Fish, squid and shrimp are mentioned in all sites as its food source (shrimp is cited relatively more often) (Table 2). In the NE, this species is cited as living in the mud, in the SE as migratory (‘andarilho’) and living in sandy substrates, and in the S as located in the water column (‘boiado’) (Table 3).
17. * Conger orbignianus* Valenciennes, 1837 (Argentine conger)
  This species is called caramuru, caramburu, and camburu in the NE; moreia in the SE; and cobra (snake) and congro in the S (Table 1). Fish were particularly mentioned as food, in addition to shrimp and squid (Table 2). Rocky substrates were mentioned in all areas (Table 3).
18. * Sphoeroides dorsalis* Longley, 1934 (Marbled puffer)
  Called baiacu in all sites, the binomial baiacu guima is used in the NE, and baiacu arara is used in the SE (Table 1). One fisherman used baiacu amarelo in the south. Its diet is noted to be shrimp in the NE, squid and fish in the SE, and is undefined in the S (Table 2). The habitat is also not well defined by fishers, with heterogeneous citations (Table 3).
19. * Bodianus ruffus* (Linnaeus, 1758) (Spanish hogfish)
  This species is called plantucano or pantucano, soldado, or Zé pereira in the NE and budião in the SE and S; two fishers called this fish budiao papagaio (Table 1). Shrimp is the most important food item mentioned, in addition to fish and mud (NE); at the other sites, most fishers did not recognize this species (Table 2). At all sites, fishers mentioned rocky substrates for this species (Table 3).
20. * Gymnothorax funebris* Ranzani, 1839 (green moray)
  As with item 17 (*Conger*), *G. funebris* is called caramuru, caramburu, or camburu in the NE and mostly moreia in the SE and S (Table 1) (Fishers appear to place the two snake-shaped fish species from the genera *Gymnothorax* and *Conger* into the same folk genus, moreia). Fish were mentioned as its primary food item at all sites, except in the S, where fishers did not recognize this fish (Table 2). Rocky substrates were mentioned in all areas (Table 3).
21. * Spheroides spengleri* (Bloch, 1785) (bandtail puffer)
  Like item 18 (*S. dorsalis*), this fish is called baiacu or baiacu guima (NE), baiacu arara (SE) and baiacu amarelo (S) (Table 1). Fishers identified shrimp as its food in the NE but fish and squid as its food in the SE (this species was not recognized in the S) (Table 2). Designations of its habitat were heterogeneous: fishers in the SE designated sandy shallows (Table 3).
22. * Mycteroperca acutirostris* (Valenciennes, 1828) (comb grouper)
  Badejo was the name given at all sites, and badejo preto in the S by 2 fishers (Table 1). Fishers from the NE cited its major food item as fish; in the SE, shrimp was the most important item mentioned, and in the S, both items (Table 2). This species is considered a rocky fish at all sites (Table 3).
23. * Rhinobatos horkelii* (Muller & Henle, 1841) (Brazilian guitarfish)
  This species is referred to as viola or cação viola or arraia viola (but just viola in the NE) (Table 1). It has the same name as another species in this study, *R. percellens* (no. 15). Shrimp was the item mentioned as food for this species at all sites (Table 2). According to fishers, this species lives in the mud (NE) and on the sandy bottom (SE and S). Gravel was also mentioned in the SE (Table 3).
24. * Seriola lalandi* Valenciennes, 1833 (yellowtail amberjack)
  This species was not well recognized in the NE: many fishers called it the same name as the bluefish; some called it olhete or guaraiuba. In the SE and S, it was called olhete (Table 1). Shrimp, fish and squid are the food items mentioned for this species (Table 2). Fishers referred to it as a rocky fish that lives in the water column and migrates (Table 3).
25. * Euthynnus alleteratus* (Rafinesque, 1840) (little tunny)
  This species is called bonito in the NE and SE. In the south, it is called cavala and bonito (fishers in the south may have confused this species with *Scomberomorus cavalla*). Binomials are bonito-cachorro and cavala cachorro (one fisherman each) (Table 1). Fish were the primary food item cited by fishers at all sites (Table 2). Fishers said that this species lives in the water column and is migratory (Table 3).
26. * Menticirrhus americanus* (Linnaeus, 1758) (southern kingcroaker)
  Papa-terra was the name given to this species by most fishers at all sites (Table 1). Shrimp, crustaceans and, secondarily, fish were the food items mentioned (Table 2). Fishers said this species lives in the mud (NE), sand (SE) and bottom (S) (Table 3).
27. * Dasyatis guttata* (Bloch & Schneider, 1801) (longnose stingray)
  This species is called arraia in the NE and arraia or arraia manteiga in the SE and S of Brazil (Table 1). Fishers said this species eats fish and shrimp in the NE and fish, squid and shrimp in the SE and S (Table 2). Its habitat was reported to be mud in the NE and sand, gravel and living on the bottom in the SE and S (Table 3).
28. * Gymnothorax ocellatus* Agassiz, 1831 (ocellated moray)
  Called mututuca or caramuru or camburu in the NE and moréia with the binomial moreia de fogo in the SE and S (Table 1), this species feeds primarily on fish and shrimp (NE) and fish and squid (S), according to fishers (Table 2). Fishers reported this species living in rocky or muddy substrates in the NE and rocky substrates and the sea bottom in the SE and S (Table 3).
29. * Trichiurus lepturus* Linnaeus 1758 (largehead hairtail)
  This species is called espada in all areas (Table 1) and is reported to feed particularly on fish but also on shrimp and squid (Table 2). Its habitat was cited as mud in the NE and sand or rock in the SE and S (Table 3).
30. CONTROL: *Pseudoplatystoma fasciatum* (Surubim, Pintado)
  This fish species was added to the study to check if fishers would reliably say that they do not know it (as marine fish). Moreover, it was a form to check how pictures were working in terms of fish recognition.
  Fishers properly recognized this species as a freshwater fish in all areas. Some fishers were aware of its name, particularly in the NE; most respondents did not recognize this fish in the S (Table 1). Most respondents did not know its feeding habitats but recognized it as a freshwater fish (Tables 2 and 3). In NE, some fishers recognize this fish because similar species could be found in the São Francisco River.
31. * Mycteroperca bonaci* (Poey, 1860) (black grouper)
  These fish are called pirambeba or badejo in the NE and badejo in the SE and S (Table 1). This species is said to feed primarily on fish in all areas but also shrimp and squid in the SE and S (Table 2). Fishers from all areas identified this species as a rocky fish (Table 3).
32. * Epinephelus marginatus* (Lowe, 1834) (dusky grouper)
  Garoupa is the primary name given in all areas (Table 1). Fishers reported this species as feeding particularly on fish but also on squid; crustacea was mentioned, by a few respondents (Table 2). This species is considered a rocky fish in all areas (Table 3).
33. * Mugil curema* Valenciennes, 1836 (white mullet)
  This species is called tainha in the NE and primarily paraty in the SE (but also tainha) and primarily tainha in the S (but also paraty) (Table 1). These fish feed on mud (NE), sand, mud or slime (SE and S). Because this species is caught by nets and not with a bait, it is probably difficult for fishers to know their diet. Heterogeneous answers were recorded in all areas, including living in both sea and rivers, being a migratory fish and living in the water column.
34. * Lutjanus cyanopterus* (Cuvier, 1828) (cubera snapper)
  This snapper is called caranha in the NE and vermelho and vermelho-caranha or caranha in the SE and S (Table 1). This species feeds on fish, shrimp and squid (SE and south) (Table 2). Fishers designated this species a rocky fish in all areas (Table 3).
35. * Caranx latus* Agassiz, 1831 (horse-eye jack)
  This species is called graçaim, xaréu or cabeludo in the NE, faqueco and xerelete in the SE, and Xaréu in the S (Table 1). According to fishers, this species feeds on shrimp, fish and squid (all areas) (Table 2). Fishers mentioned this species as living in rocky substrates but also in the water column (Table 3).
36. * Scomberomorus brasiliensis* Collette, Russo & Zavala-Camin, 1978 (Spanish mackerel)
  This fish is primarily called cavala in the NE (some called it sororoca) and primarily sororoca in the SE and S (although some respondents also called this fish cavala) (Table 1). Fishers most likely did not distinguish between the two species of *Scomberomorus* (*brasiliensis* and *cavalla*). Fish is the most important food item mentioned by fishers in all areas (Table 2), and fishers reported this species to be a migratory fish (‘andarilho’) that lives in the water column (‘boiado, aboio’) (Table 3).
37. * Aluterus monoceros* (Linnaeus, 1758) (unicorn leatherjacket filefish)
  This fish is called peixe folha or peixe rato in the NE, chinelo, perua or cangulo in the SE, and peixe porco and peixe porco branco (or cangulo) in the S (Table 1). Fishers reported that this species feeds on shrimp and squid primarily and on fish secondarily (Table 2). Diverse answers regarding the habitat of this fish were mentioned in the NE; in the SE, this species is considered to be a rocky fish and in the S to be living on the sea bottom (Table 3).
38. * Aluterus schoepfii* (Walbaum, 1792) (orange filefish)
  The responses for this species were nearly identical to 37 (Table 1). Responses for food and habitat were also nearly identical (Tables 2 and 3).

### Recognition and generic ranks

Some species were not well recognized by fishers, including *Stegastes leucostictus* at all three sites (no. 3); 41 fishers (of 92) did not know this fish. The two species of *Bodianus* (*pulchellus and rufus*) were not well recognized by fishers from the SE and S (27 fishers of 58), nor was *Gymnothorax ocellatus* (11 fishers in the SE).

The generic rank is the most important because most names in this study use primary lexemes. One important observation is that the results show that fish are not distinguished by species, but only by genera or upper rankings. In all cases in which we offered pictures of different species, fishers responded with the same generic name (in a few cases with specific names – binomials) as follows:

- *Aluterus monoceros and A. schoepfii*: Both species are called peixe folha or peixe rato in the NE; chinelo, perua or cangulo in the SE; and peixe porco, peixe porco branco and cangulo in the S.
- *Bodianus pulchellus* and *B. rufus*: These species are called plantucano or pantucano in the NE and budião in the SE (not recognized in the S).
- *Bothus ocellatus* (eye flounder) and *B. robinsi* (two-spot flounder): These species are called aramaçá in the NE and linguado, solha or soia in the SE and S.
- *Centropomus parallelus* and *C. undecimalis*: These species are called robalo at all sites.
- *Caranx crysos and C. latus*: Fishers from the NE distinguish these species as guaricema (*C. crysos*) and graçaim (*C. latus*); in the SE, these species are called faqueco (referring to *C. latus*) and particularly xerelete and xareu in the S.
- *Gymnothorax funebris* and *G. ocellatus*: These species are called caramuru, caramburu or camburu in the NE and mostly moreia in the S. These species are also called the same name as the other genera (*Conger*, represented here by *Conger orbignianus*, which is a different genera).
- *Rhinobatos percellens* and *R. horkelii*: These species are called viola, arraia viola or cacao viola in the areas studied.
- *Sphoeroides dorsalis* and S*. spengleri*: These species are called baiacu at all sites, baiacu guima in the NE and baiacu arara in the SE.
- *Lutjanus synagris* and *L. cyanopterus*: In the NE, fishers differentiate amongst species. The first is named ariocó and the second, caranha or vermelho caranha. In the SE, they are called vermelho cioba or caranha although the fishers in these areas did not distinguish between these fish as fishers from the NE did. Significantly, snappers are relatively more important in the NE fisheries than in the SE and S [15].
- *Mycteroperca bonaci* and *M. acutirostris*: These species are called badejo at all sites.

### Grouping fish (cousins, same family, relatives)

**Fig. 3:**
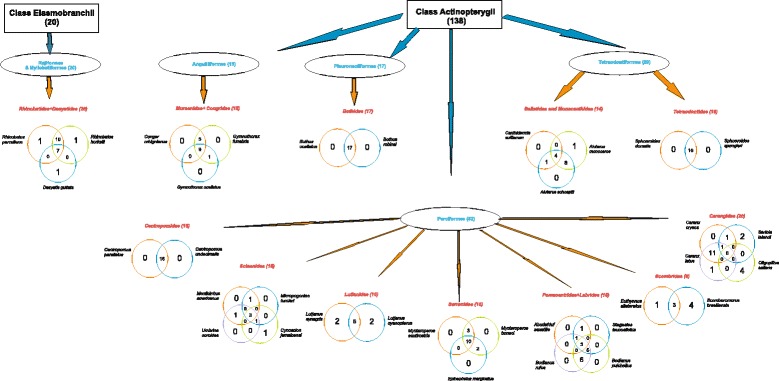
Clusters made by fishers based on their perceptions as ‘cousins’ or ‘relatives’ in the Northeast Brazil (Itacimirim and Porto do Sauípe, Bahia State)

**Fig. 4:**
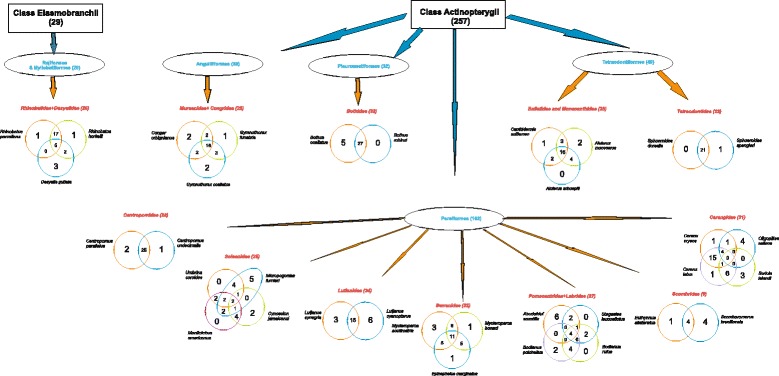
Clusters made by fishers based on their perceptions as ‘cousins’ or ‘relatives’ in the Southeast Brazil (Copacabana and Itaipu, Rio de Janeiro State)

**Fig. 5:**
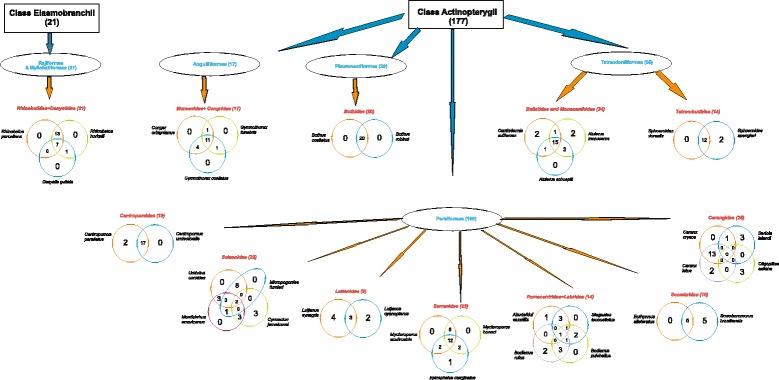
Clusters made by fishers based on their perceptions as ‘cousins’ or ‘relatives’ in the South of Brazil (Pântano do Sul, Florianópolis, Santa Catarina State)

Most fishers from the SE and S grouped fish according to the labels of ‘relatives’, ‘cousins’ or from the same ‘family’; interviewees forming grouping were more represented in the SE and S than in the NE (Table 4). The species that appear in groups are shown in decreasing order in Table 5. Groups are homogeneous in all areas (Figs. 3, 4 and 5), and many groups formed by fishers corresponded to scientific families (Centropomidae, Sciaenidae, Lutjanidae, Serranidae, and Carangidae, amongst others).

**Table 4:** Total numbers of interviewed fishers in localities along the Brazilian coast and number of groups (clusters)

Local	Interviewers	Interviewers making fish clusters (called cousins, relatives by fishers)	Number of grouping
NORTHEAST:	34	18	155
Porto Sauipe	22	6	57
Itacimirim	12	12	98
SOUTHEAST:	35	32	282
Itaipu	12	12	100
Copacabana	23	20	182
SOUTHERN: Florianópolis	23	22	198
Total All	92	72	635

**Table 5:** Order of fish presentation to fishermen and the number of citations per species (sorted by frequencies of mentioning) along the Brazilian coast (detailed methods)

ID_fish	Fish species – Author of photograph	Northeast	Southeast	Southern	Total
2	*Bothus ocellatus – D. Flesher*	17	32	20	69
23	*Rhinobatos horkelii – I. Sazima*	18	25	21	64
9	*Bothus robinsi – D. Flesher*	17	27	20	64
15	*Rhinobatos percellens – I. A Martins*	19	23	20	62
8	*Centropomus parallelus – U. Krumme*	16	27	19	62
37	*Aluterus monoceros – J. E. Randall*	13	25	21	59
12	*Centropomus undecimalis – J. F. Camrrubi*	16	26	17	59
31	*Mycteroperca bonaci – J. E. Randall e RAM Silvano*	15	23	20	58
22	*Mycteroperca acutirostris - A.A Bertoncini*	13	25	20	58
38	*Aluterus schoepfii - Jamarc*	13	22	20	55
35	*Caranx latus - A. Carvalho filho*	13	22	18	53
17	*Conger orbignianus - Inidep*	14	22	16	52
21	*Sphoeroides spengleri – D. Flesher*	16	22	14	52
14	*Caranx crysos – D. Flesher*	13	22	17	52
32	*Epinephelus marginatus – J. E. Randall*	12	22	17	51
20	*Gymnothorax funebris – E. Hofinger*	15	22	13	50
18	*Sphoeroides dorsalis – D. Flesher*	16	21	12	49
28	*Gymnothorax ocellatus – L.O. Duarte*	10	23	16	49
4	*Canthidermis sufflamen – P.M.N. C. Duarte*	5	22	19	46
11	*Micropogonias furnieri - A. Carvalho Filho*	13	17	14	44
10	*Umbrina coroides - A. Carvalho Filho*	13	11	16	40
6	*Bodianus pulchellus – J. E. Randall*	14	17	8	39
26	*Menticirrhus americanus – D. Flesher*	13	13	12	38
19	*Bodianus rufus – J. Venier*	15	16	7	38
34	*Lutjanus cyanopterus – R. Wiggers*	8	21	5	34
7	*Lutjanus synagris – I. A. Martins*	8	18	7	33
3	*Stegastes leucostictus – R. Patzner*	10	15	8	33
27	*Dasyatis guttata – Jamarc*	8	10	8	26
36	*Scomberomorus brasiliensis – L. A. Cada*	7	8	10	25
1	*Abudefduf saxatilis – R. Patzner*	5	13	6	24
13	*Cynoscion jamaicensis - Jamarc*	5	10	8	23
5	*Pomatomus saltatrix – B. Sarp*	3	12	5	20
24	*Seriola lalandi - Seafic*	4	5	7	16
16	*Oligoplites saliens – RAM Silvano*	4	9	3	16
25	*Euthynnus alleteratus – E. Hofinger*	4	5	5	14
33	*Mugil curema – Cenaim*	3	3		6
29	*Trichiurus lepturus – D. Flesher*	1	2	2	5
30	*Pseudoplatystoma fasciatum – K. Magalhaes*	0	0	1	1

### Hypotheses testing:

**Fig. 6:**
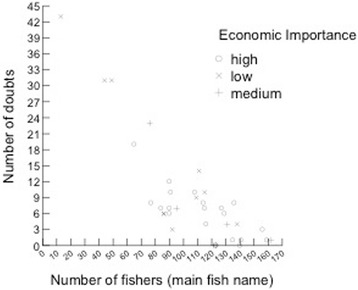
Correlation (*r* = −0.83, *n* = 37, *p* < 0.001) between the number of doubts and the number of interviewed fishers of all regions (total *n* = 161) who mentioned the most cited name of each fish species (*n* = 37) in the Atlantic Forest coast of Brazil. Numbers correspond to fish species studied listed in the Table 6

The two variables adopted to measure fishers’ knowledge, number of doubts and number of fishers mentioning the main fish name, were inversely related (*r* = −0.83, *n* = 37, *p* < 0.001), indicating that some fish species were less known and others better known by fishers (Fig. 6). The three less known fish species, such as *Stegastes leucostictus, Bodianus puchellus* and *B. rufus* all had low economic value, while most of those species well-known by fishers, such as *Centropomus parallelus* and *C. undecimalis*, had high economic value (Fig. 6).

**Fig. 7:**
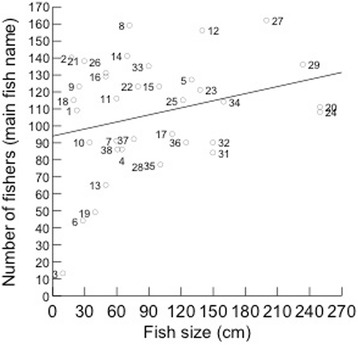
Relationship between the fish size (measures as maximum length in cm) (*r*2 = 0, *n* = 37, *p* = 0.99) and a) number of doubts; and b) number of interviewed fishers (*r*2 = 0.08, *n* = 37, *p* = 0.1) of all regions (total *n* = 161) who mentioned the most cited name of each fish species (*n* = 37) in the Atlantic Forest coast of Brazil. Numbers correspond to fish species studied listed in the Table 6

The hypothesis 1 was not confirmed: fish size was unrelated to fishers’ knowledge, either measured as number of doubts (r^2^ = 0, *n* = 37, *p* = 0.99, Fig. 7a) or as number of fishers mentioning the main fish name (*r*^2^ = 0.08, *n* = 37, *p* = 0.1, Fig. 7b).

**Fig. 8:**
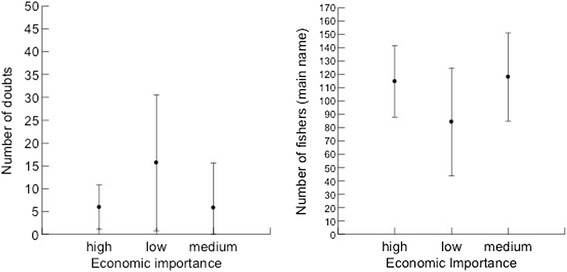
Comparison of average (± standard deviation) values of a) number of doubts (values were log10 transformed for analysis) and b) number of interviewed fishers of all regions (total *n* = 161) who mentioned the most cited name of each fish species (*n* = 37) in the Atlantic Forest coast of Brazil. Letters above bars indicate the means that were statistically different according to the Bonferroni post-hoc test

The hypothesis 2 was confirmed: fish species with low economic value showed a higher average number of doubts (*F* = 4, *n* = 37, *p* < 0.05, Fig. 8) and a lower average number of fishers who mentioned the main fish name (*F* = 3.9, *n* = 37, *p* < 0.05, Fig. 8). Interestingly, the number of doubts was statistically different only between fish species with medium and low value (Figs. 8), although the difference in average doubts between low and high value was nearly significant (*p* = 0.07, Bonferroni post-hoc test).

## Discussion

### Fish identification and biological information

This section examines the results based primarily on Fishbase and compares the literature with the fishers’s knowledge following a modification of the rationale proposed by some authors [25], as follows: GC = good concordance, MC = medium concordance, LC = low concordance, BD = badly recognized by fishers, and FK = only fish knowledge available. BD indicates that fishers were either not able to recognize the picture or they did not know the fish.

*Abudefduf Saxatilis* (no.1) (sargeant-major) is called capiacaba (NE), sargento or sargento mouro (SE) and salgo (S). The Fishbase data [28] show a diversity of names for this species in Brazil, some associated with freshwater fish (cará, acará). According to the biological description in Fishbase [28], our results indicated that fishers have strong knowledge regarding the diet and habitat of this species: “*Juveniles are common in tide pools while adults* [are] *found over shallow reef tops. Adults frequently form large feeding aggregations of up to several hundred individuals. Food items include algae, small crustaceans and fish, and various invertebrate larvae”* [28]*.***GC.**

*Bothus ocellatus* (no. 2) (eye flounder), called aramaçá (NE), linguado (SE, S) and solha (a few), has the same names as in [28]. The fishers’s description of its habitat is consistent with the following description (the diet, however, is emphasized as shrimp, not fish, by fishers): “*Inhabits sandy areas with coral rubble or seagrasses, usually near patch reefs. About one-third of the diet consists of fishes; the rest of its food is crustaceans: crabs, shrimps, amphipods, and mantis shrimps* [28]. **MC.**

*Stegastes leucostictus* (no. 3) (beaugregory), called plantucano or pantucano (NE) and poorly recognized in the SE and S (approximately half of all fishers did not recognize this fish), is cited as Gregory in Brazil [28], having its biology described as follows:

*“Adults occur in seagrass beds, coral or rocky reefs and sandy areas”. “Adults feed on algae, polychaetes, amphipods, foraminiferans and gastropods“* [28]. Its habitat is consistent with the fishers’s description, but not its diet. **LC, BR.**

*Canthidermis sufflamen* (no. 4) (ocean triggerfish) is not named (Brazilian name) in Fishbase [28]; fishers in Brazil call this fish capado (NE) and cangulo, porco, peixe and porco (SE, S). The fishers’s descriptions of habitat and diet are not consistent with Fishbase. Fishers mention deep habitats and fish and shrimp as its diet. *“Occasionally in shallow water. Solitary or in small groups in open water. Often associated with Sargassum. Feeds mainly on large zooplankton”.* [28] **LC.**

*Pomatomus saltatrix* (no. 5) (bluefish), called enchova or anchova, were consistent with the fishers in both fish diet and habitat [28]: *“They are most common along surf beaches and rock headlands in clean, high energy waters. … Feed on other fish, crustaceans and cephalopods”.****GC.*** Fishers from Brazil and Australia shared similar knowledge about the migratory behaviour of this fish, which usually moves from the south to the north [33]. According to other ethnoecological study, fishers’ knowledge about the diet of enchova agree well with biological sampling, both indicating that this fish eats mainly fish, besides shrimp and squid [20]. The name given in the NE Brazil (olhete) seem a misrecognition of this species by NE fishers, since *Pomatomus saltatrix* occur in the NE area. It is circumglobal, occurring in tropical and subtropical waters, with the exception of eastern Pacific [28].

*Bodianus pulchellus (*no. 6) (spotfin hogfish) was called the same name of the name given to *Stegastes leucostictus* (no. 3, in the NE). This fish was poorly recognized in the SE and S. Although fishers described its habitat as rocky and its diet as fish and shrimp, the fishers recognized this species as a reef fish and cited the bait the fishers use as its diet. **MD, BR.**

*Lutjanus synagris* (no. 7) (lane snapper) is called by similar names by fishers and in [28] (ariocó in the NE, vermelho cióba and vermelho caranha in the SE and S).

*“Adults are found over all types of bottoms, but mainly around coral reefs and in vegetated sandy areas. Feed at night on small fishes, bottom-living crabs, shrimps, worms, gastropods and cephalopods”* [28] **GC.**

*Centropomus parallelus* (no.8) (fat snook) is called robalo. Camurim (or camorim) is a name also found [28]; actually it is a well-known name in the NE; however, even in the NE, in this study, they called this fish robalo. *“Inhabits coastal waters, estuaries and lagoons, penetrating into freshwater. … Feeds on fish and crustaceans”* [28]*.* Both habitat and diet are similar to descriptions by fishers (sea, rivers, shrimp and fish). **GC.** In a broad survey about fishers’ knowledge on fish reproduction, interviewed fishers mentioned that this species moves between the coast and rivers or estuaries and that it spawns in coastal rivers, which agree with evidence from the biological literature [18].

*Bothus robinsi* (no. 9) (two-spot flounder) is called B. ocellatus (no. 2) by fishers. The fishers do not differentiate between these species and use the same generic name for both scientific species (polytypic genera). It *“occurs in bays, lagoons, and shallow coastal waters. … Found on soft bottoms. Feeds on crustaceans, polychaetes and mollusks”* [28]. Fishers’s descriptions are consistent with the fishbase; instead of sand, in the NE, mud is mentioned. Bahia has rocky shorelines but is also located near river mouths such as Jacuípe (Itacimirim) and Sauípe (Porto do Sauípe). **GC.**

*Umbrina coroides* (no. 10) (sand drum), mostly called corvina (also papa terra in the NE) by fishers, is the corvine [28]: “*Inhabits the surf zone along sandy beaches, but in clear water. Also occurs over muddy bottoms in estuaries and sometimes near coral reef areas. Feeds on small crustaceans washed off sand by the surf”.* The fishers´s descriptions are quite consistent with scientific knowledge. **GC**.

*Micropogonias furnieri* (no. 11) (whitemouth croaker) is called corvine (and papa terra) like *U. coroides* [28]: *“Found over muddy and sandy bottoms in coastal waters and in estuaries…; juveniles feed on benthic migratory crustaceans and sessile boring mollusks while adults are benthos-feeders and occasionally capture fish”.* We note good consistency between fishers’s knowledge and scientific knowledge. **GC.** The information provided by fishers regarding the diet and habitat agree with a biological survey, according to which this fish species eats small crustaceans in the sand bottom of coastal shores [34]. Nevertheless, the crustaceans identified in the diet of this fish by biologists [34] are possibly not the same cited by fishers.

*Centropomus undecimalis* (no. 12) (common snook) is also called robalo. Like the other *Centropomus* of this study, camurim and robalo are cited in Fishbase [28]: *“Adults inhabit coastal waters, estuaries and lagoons, penetrating into freshwater; feed on fishes (Gobiidae, Gerreidae, Engraulidae) and crustaceans (shrimps and crabs)”.***GC***.*

*Cynoscion jamaicensis* (no. 13) (Jamaica weakfish) is called pescada and goete in Fishbase [28] in addition to a variety of other names, but not samucanga.“ *Found over sand or mud bottoms from the coastline to approximately 60 m depth … Feed on fishes and crustaceans like crabs and shrimps”* [28]*.* This fish was not well recognized in the NE. **GC.**

*Caranx crysos* (no. 14) (bluerunner) has the names guaricema (from the NE) and xaréu (S) in the fishbase. Canarinho and manezinho are not in this database (although numerous diverse names appear there for Brazil). Xerelete is called Xarelete in the fishbase although those two names refer to the identical fish [28]: *A schooling species generally not far from the coast… Adults feed on fishes, shrimps, and other invertebrates”.* This species is called “andarilho” by fishers, who describe it as living within the water column. The fishers are consistent [28] with regard to diet. **GC.**

*Rhinobatos percellens* (no. 15) (Chola guitarfish) is called viola, arraia viola, and caçao viola by fishers; these names are also found in the literature, but there is no information regarding its diet or habitat [28]. In this case, fishers’s information may indicate avenues for future research. **FK.**

*Oligoplites saliens* (no. 16) (castin leatherjacket) is called guaivira (and its variants); solteira appears in the Fishbase [28]: “*Adults are found over soft bottoms of the continental shelf, often inshore and in estuaries. Also pelagic and encountered throughout the water column … May feed on plankton by ram-filtering. Juveniles feed mainly on planktonic crustaceans and chaetognaths, to a minor extent on benthic crustaceans and polychaetes, besides scales taken from larger fishes”.* The fishers´s knowledge of habitat is consistent with that description; the diet, however, except for shrimp, is not exactly as described. **MC**.

*Conger orbignyanus* (no. 17) (Argentine conger) is called the same as the species of Gymnothorax. Its eely, snake-like form may explain the identical naming of that species. Thus, we have two genera and three species under the same generic name: caramuru, caramburu, camburu (NE) and moreia (SE) (except in the S, where the fish is called congro). In Brazil, this fish is called congro [28] “*Found in shallow waters of the continental shelf … Feeds on fishes, shrimps, crabs and mollusks”.***GC.**

*Sphoeroides dorsalis* (no. 18) (marbled puffer) is called baiacu; the binomials baiacu guima and baiacu arara are not found, along other information [28]. **FK.**

*Bodianus rufus* (no. 19) (Spanish hogfish) has names not included in Fishbase, (soldado, Zé Pereira)but variants of plantucano/pantucano are found in it (pretucano) [28]. It is called the same as *B. pulchellus* (no. 6). This species is a polititypic genera. *“Adults inhabit rocky or coral reefs. Feed on brittle stars, crustaceans, mollusks, and sea urchins”* [28]. **GC.** A biological study indicates that this fish species eats invertebrates in rocky substrates [35], which partially agree with information provided by fishers.

*Gymnothorax funebris* (no. 20) (green moray) has the names caramuru and moréia [28]: “ *a benthic and solitary species occurring along rocky shorelines, reefs, and mangroves … Feeds mainly at night on fish and crustaceans”.* This information is consistent with fishers’s responses. **GC.** This snake-shaped fish is usually a food taboo, being avoided for consumption by coastal fishers in the southeast Brazilian coast [26].

*Sphoeroides spengleri* (no. 21) (bandtail puffer) is called the same name as 18, S. dorsalis, by fishers, except in the S, where baiacu amarelo is added. It can be called baiacu and baiacu mirim and baiacu pinima [28]: *“abundant in all inshore habitats where there is adequate cover, such as seagrass beds and reef flats. Feeds on mollusks, crustaceans and echinoderms”.* Fishers mentioned fish and shrimp as food and shallow waters, information that is partially consistent with the literature. **MC.**

*Mycteroperca acutirostris* (no. 22) (comb grouper) is called badejo; binomials are mentioned in the literature [28] that were not mentioned by fishers. As described [28], “ *Adults are found on rocky bottoms with high relief. Probably feeds on plankton (no information is available on the food of this species)”.* Fishers referred to this species as a rocky fish that feeds on fish and shrimp. **FK.**

*Rhinobatos horkeli* (no. 23) (Brazilian guitarfish) are referred [28] by the same names as no. 15 (*R. percellens)*: “*Found from the coast line to the continental edge. Feeds on crustaceans, cephalopods, polychaetes and small fishes”.*The fishers mentioned shrimp as food and the habitat as a muddy or sandy bottom. There is relatively little knowledge regarding this species. **GC/FK** (here we stress the importance of the fishers’s knowledge, where biological information is scarce).

*Seriola lalandi* (no. 24) Yellowtail amberjack. Olhete is the name used in this study and also in the literature [28]: *“Adults are benthopelagic in coastal and oceanic waters, off kelp beds and rocky areas, sometimes entering estuaries … can be found near rocky shores, reefs and islands … Adults feed on small fish, squid and crustaceans”.* Fishers mentioned rocky shores as habitats and a similar diet. **GC.**). In a previous study about fishers’ knowledge on fish reproduction, many interviewed fishers do not known the reproduction of this fish (and other pelagic fishes), which raises concerns of overfishing, as the fished population may include mostly juveniles [18].

*Euthynnus alletteratus* (no. 25) (little tunny). The names the fishers used are also in [28], such as bonito and bonito cachorro; “*Found in neritic waters close inshore … is an opportunistic predator which feeds on virtually everything within its range, i.e., crustaceans, fishes (mainly clupeoid), squids, heteropods and tunicates”* [28]. **GC.**

*Menticirrhus americanus* (no. 26) (southern kingcroaker) was called papa terra by fishers. Other additional names are used for this species [28]: “*Inhabits coastal waters, usually over sandy-mud to hard sand bottoms” (*there is no information on its diet). **GC/FK.**

*Dasyatis guttata* (no. 27) (longnose stingray). The name arraia manteiga is not included in Fishbase [28], nor is its diet or substrate, except that it *“inhabits shallow waters”*. This species has commercial importance in NE Brazil, as described by these authors, in the production of gelatine, oil and in aquariums. **FK.**

*Gymnothorax occelatus* (no. 28) (ocellated moray). This species is called G. funebris (no. 20). Moreia de fogo is not found [28]: “*a solitary species commonly found on deep soft bottom areas and banks, rarely on coral reefs. Also in estuaries and lagoons. Feeds mainly on crustaceans.”* Fishers also mention fish as its diet in addition to shrimp and squid. **MC.**

*Trichiurus lepturus* (no. 29) (largehead hairtail). The name espada also appears in the literature [28] in addition to other names. “*Generally over muddy bottoms of shallow coastal waters. … Adults feed mainly on fishes and occasionally on squids and crustaceans.”* [28]. ***GC.***

The control (no. 30), *Pseudoplatystoma fasciatum,* was either recognized as a freshwater species or not recognized at all. Some fishers even mentioned about the species shown in the picture :- “that fish is not from here”. The control, shown to fishers within the set of pictures and also numbered at random, was important because allowed the observation that fishers were able to identify fish folk species (or generic ranks) through the pictures; the control was also helpful in fostering participation and interaction during the interviews. The control functioned as a form of re-assuring the fishers recognition of the pictures as well as their behaviour of being confortable in recognizing that they do not know some species.

*Mycteroperca bonaci* (no. 31) (black grouper). Badejo (or pirambeba, this later name does not appear in Fishbase [28]) is the name given. This fish is described as

*“…inhabiting rocky and coral reefs. Adults feed primarily on fishes; juveniles mainly on crustaceans”* [28]. **GC.** At Bahia State we observe that badejo is the main name for this species; when we go up northeast in Brazil, the same species is called sirigado [27].

*Epinephelus marginatus* (no. 32) (dusky grouper). In addition to garoupa, other names occur [25]: *“Adults prefer rocky bottoms. … Mainly feed on crabs and octopi; larger individuals feed on a greater proportion of fishes”*. Fishers agree with this information. **GC**. In previous ethnoecological studies in the southeast Brazilian coast, fishers mentioned that this fish species eats crustaceans and fish and can be found mainly in submerged rock outcrops and in crevices [19, 22].

*Mugil curema* (no. 33) (white mullet). A diversity of names for this fish is found [28], including parati and tainha. “*Inhabit sandy coasts and littoral pools but also occur in muddy bottoms of brackish lagoons and estuaries. Sometimes penetrate rivers. May also be found on coral reefs. Feed on microscopic or filamentous algae and small juveniles of planktonic organisms.* Fishers did not contribute information regarding its diet because most fishers do not use bait to catch this fish. **MC.** In previous surveys both in the southeast and south of Brazil, fishers mentioned that this species migrate from the sea to spawn inside coastal rivers, which contradicts the prevailing biological knowledge [18, 36].

*Lutjanus cyanopterus* (no. 34)(Cubera snapper). The fishers referred to this fish with the same name as found in the literature [28]. “*Adults are found mainly around ledges over rocky bottoms or around reefs. Feed mainly on fishes, shrimps and crabs”.***GC.**

*Caranx latus* (no. 35) (horse-eyed jack). Graçaim, cabeludo or faqueco are names not found in the Fishbase [28], which includes xareu and xarelete. “*A pelagic schooling species usually found in offshore reefs … May penetrate into brackish water and ascend rivers. Adults feed on fishes, shrimps, and other invertebrates”.***GC***.* A study of the feeding behaviour of this fish shows that it feeds both in the water column, besides following individuals of *Bodianus rufus* to opportunistically eat rocky dwelling fish [35].

*Scomberomorus brasiliensis* (no. 36) (serra Spanish mackerel). Cavala and sororoca are included in the Fishbase [28]: “*Does not migrate extensively … Feeds largely on fishes, with smaller quantities of penaeid shrimps and loliginid cephalopods”*. **GC**. We observe from the interviews that species of the genera *Scomberomorus* are not clearly differentiated by the fishers.

*Aluteros monoceros* (no. 37) (unicorn leatherjacket). Numerous names appear in the literature [28], including the names reported by fishers: cangulo, perua, and peixe porco. Peixe porco branco did not appear in the fishbase. The fishbase places its habitat “*occasionally in shallow water by steep drop-offs”. “Benthopelagic”.***LC.**

*Aluterus schoepfii* (no. 38) (orange filefish). No name found in Fishbase for Brazil [28]: “*usually found over bottoms with seagrass, sand, or mud. Feeds on a variety of plants, including algae and seagrasses”.***LC.**

This section shows that there are many regional names of fish in Brazil. Brazil is a large country, and as occurs in other languages, regional words and forms of words vary. Some fish names are quite different by region, such as those of *Bodianus* (Table 1). This example shows also a commonality of the sound of the language expressed orthographically. In the NE, Bodianus is called plantucano or pantucano; in the SE, budião (this study) and godião or gudião [3, 7]. We compared our results with the fishbase data set [28], and in that database, we also observed diverse names in Brazil. Other studies have also shown this huge diversity of fish names [37].

Robalo is a well-known name in the NE [28]; however, the name camurim was not mentioned during this study in Bahia (NE). This state is located at the edge of the Brazilian NE, in the middle of the country. The name camurim is most likely used at higher coastal latitudes, such as in Alagoas State [9].

### Fishers’s knowledge and fish usefulness

Earlier studies [3, 15, 38] have shown that fishers have extensive knowledge of target species of small-scale fisheries compared with discarded species (bycatch) or species that are not consumed nor commercialized. This observation appears valid also for this study. High local importance, represented here as fish commercialized or consumed, was linked to fishers’s knowledge either more homogeneous or more consistent with the scientific literature (Table 6). Indeed, we confirmed the hypothesis 2 and observed that fish species with low economic importance were less known by fishers.

**Table 6:** Fishermen’s knowledge relative to the scientific literature concordance [43] and relative importance of the fish

Species	Knowledge	“DNK”	Local Importance
*1. Abudefduf saxatilis*	GC	9	low
*2. Bothus ocellatus*	MC		high
*3. Stegastes leucostictus*	LC, BR	43	low
*4. Canthidermin sufflamen*	LC	6	low
*5. Pomatomus saltatrix*	GC	7	high
*6. Bodianus puchellus*	MD, BR	31	low
*7. Lutjanus synagris*	GC	10	high
*8. Centropomus parallelus*	GC	1	high
*9. Bothus robinsi*	GC		high
*10. Umbrina coroides*	GC	6	high
*11. Micropogonias furnieri*	GC	4	high
*12.Centropomus undecimalis*	GC	3	high
*13. Cynoscion jamaicensis*	GC	19	high
*14. Caranx crysos*	GC	1	high
*15. Rhinobatos percellens*	FK		medium
*16. Oligoplites saliens*	MC	6	high
*17. Conger orbignyanus*	GC	7	medium
*18. Sphoeroides dorsalis*	FK	10	low
*19. Bodianus rufus*	GC	31	low
*20. Gymnothorax funebris*	GC	14	low
*21. Sphoeroides spengleri*	MC	4	low
*22. Mycteroperca acutirostris*	FK		high
*23. Rhinobatos horkelii*	GC, FK		medium
*24. Seriola lalandi*	GC	10	high
*25. Euthynnus alleteratus*	GC	7	high
*26. Menticirrhus americanus*	GC, FK	4	medium
*27. Dasyatis guttata*	FK	1	medium
*28. Gymnothorax occelatus*	MC	23	medium
*29. Trichiurus lepturus*	GC	8	high
*30. CONTROL –Pseudoplatystoma fasciatum*	-	26	-
*31. Mycteroperca bonaci*	GC	7	high
*32. Epinephelus marginatus*	GC	7	high
*33. Mugil curema*	MC	1	high
*34. Lutjanus cyanopterus*	GC	8	high
*35. Caranx latus*	GC	8	high
*36. Scomberomorus brasiliensis*	GC	12	high
*37. Aluteros monoceros*	LC	3	low
*38. Aluteros schoepfii*	LC	6	low

*GC* Good concordance, *MC* medium concordance, *LC* low concordance, *FK* fisher’s knowledge, *BR* badly recognized. Local importance (based on local observations and literature: [27, 44]: low = not sold, seldom consumed, considered as mistura (trash); medium: not sold, consumed OR sold, not commonly consumed ; high: sold and consumed. Medicinal, but considered as mistura (trash). “*DNK*” does not know

The homogeneity of responses is an interesting indicator of fishers’s knowledge, as fishers share information. Thus, discussing species involves sharing this knowledge that is represented in the uniformity of answers. Species such as *P. saltatrix*, and the ones in the genus *Caranx* and *Mycteroperca* are such examples. Notably, and understanding the concept of usefulness that includes a venomous animal [39] we observed that information regarding the species *Sphoeroides* was scarcely consistent. Either fishers have different knowledge from scientists or have little knowledge of that species. Knowledge can be affected by environmental stimuli and the necessity to acquire the skills to be more active: in that regard, fishers’s knowledge appears to be associated with target species. In this sense, the lack of knowledge about *Sphoeroides* may be because this species is neither sold nor consumed (Table 6).

It is understandable that target species should be observed more often by fishers, in their daily fishing trips, compared to other species. In fact, to be a good marine predator, one must know the prey’s location (habitat) and know which bait to use (food items) [29]. Other authors have discussed the size of the fish as an indicator of the mental stimulus to classification and identification [5]; however, we did not observe such a tendency in the fishers in this study, as we did not confirmed our hypothesis of a positive relationship between fishers’ LEK and fish size. One aspect that should be studied in more detail is how fishers determine the diet of the fish or the bait that should be used for the fish. Although bait and diet can be correlated [20, 33], the two are not identical. More research should be conducted in that area because many responses suggested that fishers were talking about which bait they used. Overall, in terms of the information on the fish diet, given by fishers, that could be considered a minor caveat that could be transposed with a care, during interviews, about the fish diet questions made to fishers.

### Generic rank and primary lexemes

In this study, we observed that recognition is based on generic rank, and some ranks may seem polytypic, such as the ranks in which we have shown two species (*Aluterus, Bothus*, *Centropomus, Gymnothorax, and Mycteroperca*, amongst others). In other words, fishers usually did not differentiate amongst species of the same scientific genera. In that regard, we are not considering those genera as polytypic because fishers gave the same description and names for the species. Examining the most important fish families of Porto do Sauípe (abundant, consumed and sold) in detail, we observed that Lutjanidae is polytypic, including prototypes [40]. Generic names are the most common nomenclature given by fishers in this study. Similar findings occurred in studies of Atlantic Forest fishers; however, binomials appeared to be more common in those other studies but especially among riverine Amazonian fishers. [3, 6]. The commonality of generic names seem to be a general pattern in ethnotaxonomy [1]. In a comparison of the ethnotaxonomy of the Amazon fishers and the Atlantic forest fishers, we observed that the more detailed taxonomy of Amazonian fishers led those fishers to label species using binomials more often than the fishers from the Atlantic Forest coast. Such a difference was attributed to the need to differentiate amongst similar forms in the rivers; fish from the Atlantic Forest coast have quite different fish morphologies and thus do not require such detailed binomials [3].

### Fish clusters and fishers’s information on target species

The fish clusters formed by fishers are consistent with findings from earlier studies: that these groups are often related to important fish (Carangidae, Serranidae, amongst others) (Table 6) and that fishers do classify fish in groups [3, 11].

There are some general features found in this study that are important to highlight:

A. fishers do form clusters of fish species, usually hierarchically. Such clusters are often in concordance with the scientific clusters, and they are especially related to target fish.
B. Fishers of the coast of Brazil used mostly primary lexemes (generic names) for naming fish (observe that riverine fishers of the Amazon use mostly binomials [3]).
C. Fishers did not differentiate between scientific species belonging to a genus: the same folk generic name was given by fishers for two different scientific species, in all the cases pictures of two different scientific species to fishers. Therefore, we reinforce the suggestions that folkbiology universals exist, as already observed [1, 41].
D. Fishers’ information on diet and habitat: as this study shows, fishers can be very helpful in informing about the fish diet and the fish habitats. There are species in which information is scarce or absent [28], such as *R. percellens, M. acutirostris, M. americanus,* and *D.* guttata. (See other studies such as on Ilhabela fishers, where there is detailed information on the food web constructed by fishers and where it has been shown how scarce is the information for Brazilian coastal waters fishes [42]). When considering habitat, we observed how depth is important for fishers of Bahia, were ‘*the wall*’ is a frontier, but also a place where catches from deep waters are obtained. Moreover, we observed that information on habitat given by the fishers reflected local environmental variabilities. For example, in the NE (Bahia State), communities are located close to rivers mouths, and fishers mentioned more muddy bottoms, compared to the sandy bottoms mentioned in the SE and S of Brazil. Moreover, the narrow continental shelf of the NE, compared to the SE and S coasts, increases the accessibility of fishers to deeper waters, where the habitat is classified based on different depths (called “peça” or “meia-peça”, at Bahia). Target fish seem to concentrate fishers’ knowledge, as this study has statistically shown. FAO Technical paper 591 [24] suggests:

‘*Fishers have a wealth of knowledge and experience that is extremely valuable for research and management of fisheries, particularly in the case of small-scale fisheries in developing countries, where scientific data are often scarce’.*

The articles included in this FAO Technical Paper 591 [24] are from several small-scale fisheries of Latin American, and they have as one of the objectives to guide the integration of Fisher’s Knowledge to EAF (Ecosystem Approach to Fisheries). We, thus, suggest that biologists and other researchers, along with agents from environmental agencies should at least pay attention to what fishers’ says as well as to their demands.

## Conclusions

Fishers’ nomenclature follows regional names, particularly when comparing the northeast and southern coasts of Brazil (SE and S). Besides, there are also linguistic variances of the same name, along with regionalisms per area (SE and NE). Generic names are the most common, represented by primary lexemes. Fishers’ knowledge of the habitat and diet of fish appear to be particularly concerned with target species, which are species that fishers encounter most often; such knowledge can be needed to achieve good catches. Fishers must know where to locate the fish and which bait to use. Fishers also group fish, and their groupings are similar in the three areas; again, the groups cited most often are those groups that include most important fish. Finally, we can conclude that fishers showed a comparatively higher knowledge of target species compared to less important species (not consumed, or not sold, for example). Overall, fishers showed good knowledge about most of the studied fish species considering an analogy with the scientific literature and for some species fishers’ knowledge may be the sole source of information available. These results reinforce the relevance to include fishers’ knowledge in fisheries management.

### Ethics

Data of this manuscript reports on data collected in the years 2004 and 2005, based on questionnaires by which we had verbal consent from the fishers. Our study is essentially based on primary data collected during this time and not published yet, taken from the questionnaires deposited at the FIFO’s archives, labelled PEMVIMSA 005 from 2004 to 5. In this case ethics committee is not applicable. Our study does not report or involve the use of any animal or human data or tissue.

## Additional file

Additional file 1: Supplementary material. (DOCX 123 kb)

## Abbreviations

BR: badly recognized
Dnk: does not know
FK: fisher’s knowledge
GC: good concordance
LC: low concordance
MC: medium concordance
NE: Northeast
S: South
SE: Southeast
