# Supplementary material for: Fishers’ knowledge on the coast of Brazil
Source: J Ethnobiol Ethnomed. 2016 Jun 1;12:20. doi: 10.1186/s13002-016-0091-1 (PMC4888665; doi:10.1186/s13002-016-0091-1)
Supplement: Supplementary file 1 — Supplementary material. (DOCX 123 kb) [file 13002_2016_91_MOESM1_ESM.docx]

**Supplementary material: some references including fish collected and identified, along with other information for the study sites and nearby sites by authors**

**FISHERMENS’ KNOWLEDGE ON THE COAST OF BRAZIL**

**Alpina Begossi^1,2,3^, Svetlana Salivonchyk^4^, Priscila F. Lopes ^2,5^ and Renato A. M. Silvano ^2,6^**

**^1^** Capesca, NEPA, UNICAMP: Av. Albert Einstein 291, CEP 13083-852, Campinas, SP, Brazil.

^2^ Fisheries and Food Institute, [www.fisheriesandfood.org](http://www.fisheriesandfood.org)

**^3^** Ecomar, Unisanta, Rua Cesário Mota, 08, CEP 11045-040, Santos, SP, Brazil.

^4^ Institute for Nature Management, National Academy of Sciences of Belarus, 10 Fr. Skaryna Street, Minsk, 220114, Minsk, Belarus

^5^Fishing Ecology, Management and Economics Group (FEME), Dept. of Ecology, Federal University of Rio Grande do Norte (UFRN), Natal, RN, Brazil. 59078-900.

^6^ Universidade Federal do Rio Grande do Sul (UFRGS), Dept. of Ecology, CP 15007, 91501-970, Porto Alegre, RS, Brazil

In this session we list material collected and published at the sites of this study, ora t some nearby sites of the NE, SE and South of Brazil, but in particular of Porto do Sauipe and Itacimirim (Bahia State ), Itaipu (Niteroi) and Copacabana (Rio de Janeiro State), and Pântano do Sul (Santa Catarina State). From this material many fish species collected are shown, along other information on some fish species, such as diet and habitat.

Begossi A: **Local knowledge and training towards management**. *Environment, Development, and Sustainability* 2008, 10:591–603.

Begossi A, and Figueiredo, JM. **Ethnoichthyology of Southern coastal fishermen: cases from Buzios Island and Sepetiba bay** 1995, 56:710-717.

Begossi A, Camargo E, Carpi Jr S: **Os Mapas Da Pesca Artesanal - Pesqueiros E Pescadores Na Costa Do Brasil.** São Carlos: Editora Rima; 2013.

- Begossi A, Silvano R: **Ecology and Ethnoecology of dusky grouper, garoupa, [Epinephelus marginatus (Lowe, 1834)] along the coast of Brazil.** *J Ethnobiol Ethnomedicine* 2008, 4:1–20. See Table 3, where crab identification is shown for *Mycteroperca marginata* (dusky grouper).
- Begossi A, Salivonchyk LG, Araujo TB, Andreoli M, Clauzet M, Martinelli CM, Ferreira AGL, Oliveira LEC, Silvano R: **Ethnobiology and snapper conservation in the artisanal fisheries of Brazil: target species and suggestions for management**. *J Ethnobiol Ethnomedicine* 2011, **7**:11. See Table 4, showing identified stomach contents for species of Lutjanidae (fish, mollusks, and crustacean).
- Begossi, A, Lopes PFM, and Silvano RAM. 2012. **Co-Management of Reef Fisheries of the Snapper-Grouper Complex in a Human Ecological Context in Brazil.** In: G.H. Kruse, H.I. Browman, K.L. Cochrane, D. Evans, G.S. Jamieson, P.A. Livingston, D. Woodby, and C.I. Zhang (eds.), *Global Progress in Ecosystem-Based Fisheries Management.* Alaska Sea Grant, University of Alaska Fairbanks.

Begossi A, Hanazaki N, Ramos RM: **Food chain and the reasons for fish food taboos among amazonian and atlantic forest fishers (Brazil**). *Ecol Appl* 2004, 14:1334–1343.

Herbst DF, Hanazaki N: **Local ecological knowledge of fishers about the life cycle and temporal patterns in the migration of mullet (Mugil liza) in Southern Brazil**. *Neotropical Ichthyol* 2014, **12**:879–890.

- Lopes, P. F. M. 2008. Extracted and farmed shrimp fisheries in Brazil: economic, environmental and social consequences of exploitation. *Environment, Development & Sustainability* 2008, 10: 639-655.

Oliveira LEC de, Barreto Tainá, Begossi A: **Prototypes and Folk Taxonomy: Artisanal Fishers and Snappers on the Brazilian Coast**. *Curr Anthropol* 2012, **53**:789–798.

Ramires M, Clauzet M, Barrella W, Rotundo MM, Silvano RA, Begossi A: **Fishers’ knowledge about fish trophic interactions in the southeastern Brazilian coast**. *J Ethnobiol Ethnomedicine* 2015, **11**:19.

Silvano RAM: **Feeding Habits and Interspecific Feeding Associations of Caranx Latus (Carangidae) in a Subtropical Reef**. *Environ Biol Fishes* 2001, **60**:465–470.

Silvano RAM, and Begossi A. **Local knowledge on a cosmopolitan fish**

**Ethnoecology of *Pomatomus saltatrix* (Pomatomidae) in Brazil and Australia.** *Fisheries Research* 2005, ***41****: 53-79*

Silvano RAM, Begossi A. **What can be learned from fishers? An integrated survey of fishers’ local ecological knowledge and bluefish (Pomatomus saltatrix) biology on the Brazilian coast**. *Hydrobiologia* 2010, **637**: 3–18.

Silvano RAM, Begossi A: **Fishermen’s local ecological knowledge on Southeastern Brazilian coastal fishes: contributions to research, conservation, and management**. *Neotropical Ichthyol* 2012, **10**:133–147.

Silvano RAM, MacCord PFL, Lima RV, Begossi A. 2006. **When does this fish spawn? Fishermen’s local knowledge of migration and reproduction of Brazilian coastal fishes**. *Environment Biology of Fishes* 2006, **76**:371–386. Table 6, showing identified stomach contents for 184 bluefish (*Pomatomus saltatrix).*
